# Hierarchical Study of the Reactions of Hydrogen Atoms with Alkenes: A Theoretical Study of the Reactions of Hydrogen Atoms with C_2_–C_4_ Alkenes

**DOI:** 10.1021/acs.jpca.1c03168

**Published:** 2021-06-08

**Authors:** Jennifer Power, Kieran P. Somers, Shashank S. Nagaraja, Henry J. Curran

**Affiliations:** Combustion Chemistry Centre, School of Chemistry, Ryan Institute, MaREI, National University of Ireland, Galway, Galway H91TK33, Ireland

## Abstract

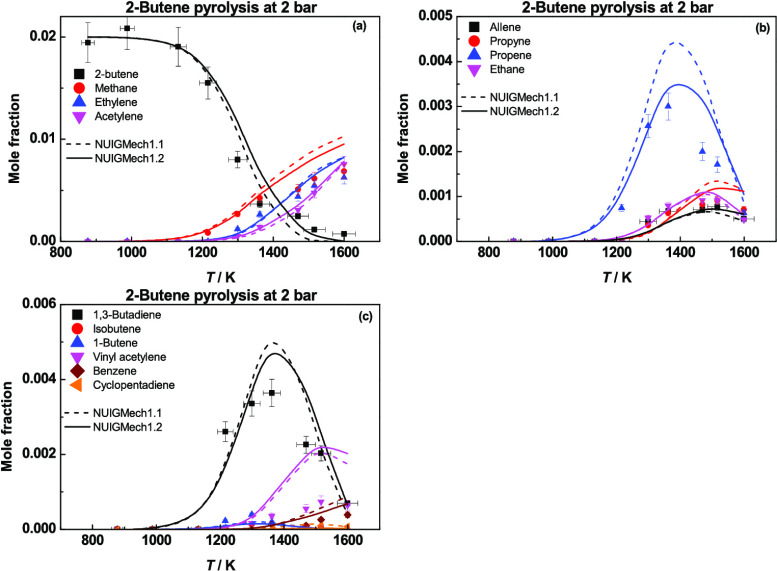

The present study complements our previous studies of the reactions of hydrogen atoms with C_5_ alkene species including 1- and 2-pentene and the branched isomers (2-methyl-1-butene, 2-methyl-2-butene, and 3-methyl-1-butene), by studying the reactions of hydrogen atoms with C_2_–C_4_ alkenes (ethylene, propene, 1- and 2-butene, and isobutene). The aim of the current work is to develop a hierarchical set of rate constants for Ḣ atom addition reactions to C_2_–C_5_ alkenes, both linear and branched, which can be used in the development of chemical kinetic models. High-pressure limiting and pressure-dependent rate constants are calculated using the Rice–Ramsperger–Kassel–Marcus (RRKM) theory and a one-dimensional master equation (ME). Rate constant recommendations for Ḣ atom addition and abstraction reactions in addition to alkyl radical decomposition reactions are also proposed and provide a useful tool for use in mechanisms of larger alkenes for which calculations do not exist. Additionally, validation of our theoretical results with single-pulse shock-tube pyrolysis experiments is carried out. An improvement in species mole fraction predictions for alkene pyrolysis is observed, showing the relevance of the present study.

## 1 Introduction

Alkenes are important intermediates formed during the oxidation and pyrolysis of larger alkanes and are key components of hydrocarbon fuels. An understanding of their combustion chemistry is therefore important in our understanding of hydrocarbon fuel combustion. The reactions of Ḣ atom across the C=C double bond play an important role in controlling experimental high-temperature ignition delay times (IDTs), flame speeds, and species profiles measured as a function of temperature and/or time in jet-stirred and flow reactors.^1−3^

In the current work, the reactions of Ḣ atoms with C_2_–C_4_ alkenes are studied, while the reactions of Ḣ atom addition to C_5_ alkenes were studied previously.^4,5^ There have been a number of theoretical and experimental studies of Ḣ atoms with C_2_–C_4_ alkenes.^17−16^ This study aims to complement these by providing a comprehensive hierarchical set of rate constants for Ḣ atom addition and abstraction potential energy surfaces (PESs), including their chemically activated pathways for C_2_–C_5_ alkenes, determined at the same levels of theory. By having a consistent set of rate constants for C_2_–C_5_ alkenes + Ḣ atoms calculated at the same level of theory, our results help constrain available models and the development of recommended rate constants, which provide a tool for use in mechanisms of larger alkenes for which calculations do not exist in the literature.

Ethylene is the smallest alkene in our series and has been extensively studied.^17,6−16^ Miller and Klippenstein^17^ studied the kinetics of Ḣ + C_2_H_2_ and Ḣ + C_2_H_4_, including their reverse dissociation reactions using variational transition state theory (VTST) and a two-dimensional (2D) master equation. Matsugi^16^ performed direct trajectory calculations on Ċ_2_H_5_ radical dissociation and discovered a reaction pathway that directly eliminates H_2_ from Ċ_2_H_5_, leading to the formation of vinyl (Ċ_2_H_3_) radicals. The resulting Ċ_2_H_3_ radicals can dissociate to C_2_H_2_ + Ḣ. They suggest that this may be an explanation for the unexpectedly slow Ḣ atom formation previously observed in photodissociation experiments of Ċ_2_H_5_ radicals.^21,22^ Barker et al.^6^ studied the reaction of Ḣ + C_2_H_4_ as a function of He pressure at room temperature with three experimental techniques: (i) a discharge flow system with Lyman-α photometry, (ii) a time-resolved Lyman-α photometric system, and (iii) a discharge flow system with time-of-flight mass spectrometry. Rate constants were obtained in both excess ethylene and hydrogen environments, and an experimental value for the third-body recombination coefficient for Ḣ + ĊH_3_ (+M) was obtained.

Michael et al.^12^ used Lyman-α photometry to obtain the pressure dependence of the Ḣ + C_2_H_4_ reaction at room temperature. Through computer simulation analysis, the rate constants were adjusted for Ḣ atom depletion in reactions subsequent to the initial reaction. Experiments at high pressures of He permitted extrapolation to the high-pressure limiting rate constant. Lee et al.^9^ experimentally measured the rate constant for the Ḣ + C_2_H_4_ reaction as a function of temperature (198–320 K) at high pressures of Ar bath gas using the flash photolysis-resonance fluorescence technique. Sugawara et al.^14^ measured the high-pressure limiting rate constants of Ḣ and Ḋ atom addition to C_2_H_4_, C_2_H_3_D, C_2_D_4_, C_2_H_2_, and C_2_D_2_ in the temperature range 206–461 K using pulse radiolysis-resonance absorption.

Pacey et al.^13^ performed pyrolysis experiments on ethane at 902 K and concentrations of 1.8 × 10^–4^–4.5 × 10^–3^ mol L^–1^ in a flow system. Rate constants for the reactions Ċ_2_H_5_ + Ċ_2_H_5_ ↔ C_4_H_10_ and Ċ_2_H_5_ + Ċ_2_H_5_ ↔ C_2_H_6_ + C_2_H_4_ were determined. Moreover, pressure-dependent rate constants for C_2_H_6_ ↔ ĊH_3_ + ĊH_3_ and Ċ_2_H_5_ ↔ C_2_H_4_ + Ḣ were determined using a unimolecular reaction rate theory. Lightfoot et al.^10^ measured the rate constant of the reaction Ḣ + C_2_H_4_ ↔ Ċ_2_H_5_ as a function of temperature and pressure, over the temperature and pressure ranges 285–604 K and 50–600 Torr, respectively, using laser flash photolysis/resonance fluorescence, with helium diluent.

Feng et al.^7^ investigated the unimolecular decomposition of Ċ_2_H_5_ radicals in helium over the temperature and pressure ranges 876–1094 K and 0.8–14.3 Torr, respectively, in time-resolved experiments. The reaction was isolated for a quantitative study in a heated tubular reactor coupled to a photoionization mass spectrometer. Hanning-Lee et al.^8^ studied the reaction Ḣ + C_2_H_4_ ↔ Ċ_2_H_5_ at 800 K in He. Exciplex laser flash photolysis at 193.3 nm of ethene–helium mixtures was used to generate Ḣ atoms, which were detected using time-resolved resonance fluorescence. Rate coefficients for the forward and reverse reactions were deduced from measurements of the equilibrium constant and relaxation rate coefficient at nine pressures in the range 97–600 Torr. More recently, Yang et al.^15^ investigated the decomposition of ethyl iodide and subsequent dissociation of ethyl radicals behind incident shock waves in a diaphragm-less shock tube using laser schlieren (LS) densitometry (1150 ≤ *T* ≤ 1870 K, and 55 ≤ *p* ≤ 123 ± 3 Torr).

Fewer studies exist for the reactions of Ḣ atoms with propene and the butene isomers. Experimental studies of Ḣ atoms with propene include refs (18−19, 23−32), with the most recent one by Chen et al.^20^ studying the temperature and pressure dependence of the product branching ratio of the Ḣ + propene reaction. This was done behind reflected shock waves in a diaphragm-less shock tube using the Ḣ-ARAS technique in the temperature range 1065–1306 K at 1 and 2 bar. Quantum chemistry calculations were also performed at the CCSD(T)/CBS//CCSD/6-311++G(3df,2p) level of theory. The predicted high-pressure limiting rate constant ratio for terminal versus nonterminal addition agrees well with that reported by Manion et al.^19^ for the analogous reaction of Ḣ atoms with butene. Both Chen et al.^20^ and Manion et al.^19^ state that their predicted branching ratio for terminal versus nonterminal addition differ from that calculated by Miller and Klippenstein who studied the dissociation of propyl radicals and other reactions on the Ċ_3_H_7_ PES.^18^ With minor adjustments to several of the barrier heights, Miller and Klippenstein showed excellent agreement between their theoretical values and experimental results available in the literature over a wide range of conditions.

Manion and Awan^19^ investigated the kinetics of terminal and internal Ḣ atom addition to 1-alkenes. Single-pulse shock tube methods were employed to thermally generate Ḣ atoms, and their reactions with 1-butene were investigated over the temperature and pressure ranges 880–1120 K and 145–245 kPa, respectively. Relative and absolute rate constants for the displacement of methyl and ethyl radicals by Ḣ atoms were determined and related to the high-pressure limiting rate constant for Ḣ atom addition to the terminal and internal sites of 1-butene. It was found that addition to the terminal site is favored by a factor of 2.6 ± 0.4 at 1000 K. These results were combined with data from lower temperatures and used by Manion and Awan to derive rate constants in the temperature range 220–2000 K. They state that these branching ratio expressions should approximate the behavior of other unbranched 1-olefins and can thus be used as estimates for unstudied 1-olefins in detailed kinetic models describing pyrolysis and combustion conditions. A factor of 3 discrepancy was noted in the branching ratio for terminal to internal Ḣ atom addition by comparing their current experimental results with the theoretical study,^18^ and they suggest that the difference observed is well outside the experimental errors of their study and any expected differences for 1-butene.

Wang et al.^33^ studied the reaction kinetics of H-atom abstraction from C_4_–C_6_ alkenes by Ḣ atoms and ĊH_3_ radicals using the G4 composite method with CTST and Eckart tunneling corrections. The study provides the first systematic report on the key initiation abstraction reaction classes for alkenes with Ḣ atoms and ĊH_3_ radicals. However, large discrepancies are observed between the Wang et al.^33^ calculations and those already present in the literature and calculated in this work.

Nagaraja et al.^32^ performed a single-pulse shock-tube study on the pyrolysis of 2% C_2_–C_6_ 1-alkenes at 2 bar in the temperature range 900–1800 K, with reactant intermediate and product species obtained and quantified using gas chromatography–mass spectrometry analysis.

One of the aims of the present study is to investigate the ratio of terminal to internal Ḣ atom addition to C_2_–C_5_ alkenes taking into account our past studies^4,5^ of the C_5_ alkenes since discrepancies remain in the literature. Rate constant recommendations for Ḣ atom addition, abstraction, and alkyl radical decomposition reactions will also be made and should serve as a useful tool for their use in mechanisms for larger alkenes where calculations do not exist.

Section 2 describes the computational methods employed in the current work, and Section 3 presents the theoretical results including comparisons with literature studies, where available (Table 1). Section 4.0 presents our simulation results compared to the shock tube pyrolysis experiments of Nagaraja et al.^32,35^

**Table 1:** Summary of Experimental and Theoretical Studies Relevant to C_2_–C_4_ Alkenes + Ḣ

year	author	reaction(s)	*T* (K)	*p* (kPa)	method
2020	Nagaraja et al.^32^	pyrolysis of C_2_–C_6_ 1-alkenes	900–1800	200	single-pulse shock tube (SPST)
2020	Chen et al.^20^	Ḣ + C_3_H_6_	1065–1306	100–200	Ḣ-ARAS/shock-tube CCSD(T)/CBS//CCSD/6-311++G(3df,2p)
2018	Wang et al.^33^	C_4_–C_6_ alkenes + Ḣ and ĊH_3_			G4 composite method
2015	Manion et al.^19^	Ḣ + C_4_H_8_-1	880–1120	145–245	single-pulse shock tube (SPST)
2013	Matsugi et al.^16^	photodissociation of Ċ_2_H_5_			direct trajectory calculations ωB97X-D/6-31+G(d,p)
2013	Miller et al.^18^	dissociation of propyl radicals and other reactions on Ċ_3_H_7_ potential		0–HPL	CCSD(T)/cc-pVTZ MP2/6-311++G(d,p)
2012	Yang et al.^15^	decomposition of ethyl iodide/dissociation of Ċ_2_H_5_ radicals	1150–1870	7.3–16.4	diaphragm-less shock tube/laser schlieren (LS) densitometry
2011	Rosado-Reyes et al.^28^	Ḣ + C_3_H_6_	922–1200	150–340	single-pulse shock tube (SPST)
2004	Miller et al.^17^	Ḣ + C_2_H_2_ and C_2_H_4_	300–2000	>0.13/HPL	variational transition state theory (VTST), 2D master equation
1993	Hanning-Lee et al.^8^	Ḣ + C_2_H_4_	800	12.9–80.0	exciplex laser flash photolysis/time-resolved resonance fluorescence
1993	Feng et al.^7^	unimolecular decomposition of Ċ_2_H_5_	876–1094	0.1–1.9	heated tubular reactor/to a photoionization mass spectrometer
1993	Seakins et al.^23^	iĊ_3_H_7_ decomposition	720–910		laser flash photolysis/photoionization mass spectrometry
1992	Tsang^36^	database for hydrocarbon pyrolysis			estimate
1992	Hidaka et al.^29^	thermal decomposition of C_3_H_6_	1200–1800		laser kinetic absorption spectroscopy/GC
1991	Tsang^37^	database for hydrocarbon pyrolysis			estimate
1989	Löser et al.^30^	Ḣ atom abstraction by allyl radicals from hydrocarbons			BSBL
1987	Lightfoot et al.^10^	Ḣ + C_2_H_4_	285–604	6.7–80.0	laser flash photolysis/resonance fluorescence
1986	Munk et al.^25^	iĊ_3_H_7_ and iĊ_3_H_7_O_2_	298	101	UV absorption/pulse photolysis
1984	Pacey et al.^13^	pyrolysis of C_2_H_6_	902	HPL	flow system
1982	Watanabe et al.^27^	Ḣ + C_3_H_6_	200–500		pulse radiolysis-resonance absorption
1982	Harris et al.^31^	Ḣ + C_3_H_6_/C_4_H_8_	298–455		flash photolysis-resonance fluorescence
1981	Sugawara et al.^14^	Ḣ- and D-atom addition to C_2_H_4_, C_2_H_3_D, C_2_D_4_, C_2_H_2_, and C_2_D_2_	206–461		pulse radiolysis-resonance absorption
1978	Lee et al.^9^	Ḣ + C_2_H_4_	198–320	0.13	flash photolysis-resonance fluorescence (FP-RF) technique
1973	Michael et al.^12^	Ḣ + C_2_H_4_			Lyman α photometry
1972	Kerr et al.^24^	evaluated kinetic data on gas-phase addition reactions			
1971	Kurylo et al.^26^	Ḣ + C_3_H_6_	298		resonance fluorescence of Lyman α radiation
1970	Barker et al.^6^	Ḣ + C_2_H_4_			discharge flow system with Lyman-α photometry, time-resolved Lyman-α photometric system, and discharge flow system with time-of-flight mass spectrometry

## 2 Computational Details

### 2.1 Electronic Structure Calculations

As mentioned earlier, we have employed the same methods here as those used in our previous studies^4,5^ to carry out all electronic structure calculations; thus, the description here is brief. All calculations carried out using Gaussian 09^38^ and Gaussian 16^39^ conformational searches were performed, with the resulting lowest-energy conformer optimized at the ωB97XD^40^/aug-cc-pVTZ^41^ level of theory. A harmonic frequency analysis was simultaneously performed at the same level of theory to verify the nature of each stationary point.

Low-frequency torsional modes were treated via relaxed PES scans in 10° increments with the ωB97XD/6-311++G(d,p)^40^ method, with the potential energies as a function of dihedral angle used as input for a one-dimensional (1D) hindered rotor approximation as implemented in the Master Equation System Solver (MESS).^42^

To compute reaction barrier heights, single point energies for minima and transition states were calculated at the CCSD(T)/cc-pVXZ and MP2/cc-pVXZ (where X = D, T, and Q) levels of theory. The resulting energies were extrapolated to the complete basis set (CBS) limit using the following formula (eq 1):^43,44^
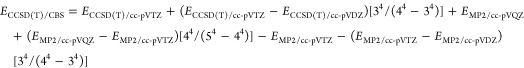
1The T_1_ diagnostic for minima and transition state species is ≲ 0.03, indicating that single reference methods to describe the wave function are appropriate.^43^ However, for the Ċ_2_H_3_ radical well and the transition states of Ḣ atom addition to and abstraction from C_2_H_4_, the T_1_ diagnostics are 0.04, 0.038, and 0.352, respectively. As a result, for the C_2_ and C_3_ reaction systems, ROCCSD(T)/aug-cc-pVXZ (where X = T and Q) single point energies were also calculated since they were computationally achievable. The energies were extrapolated to the CBS limit using eq 2:
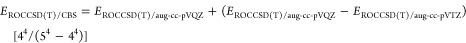
2with the resulting T_1_ diagnostics falling below 0.03. The largest difference in energy barriers as a result of using the two formulas was for H-atom abstraction from the primary vinylic sites of C_2_H_4_ and C_3_H_6_, where differences of 1.57 and 1.39 kJ mol^–1^, respectively, were observed. These differences in energy barriers increased the rate constants for these reactions by factors of 1.87 and 1.71 at 298 K.

### 2.2 Thermochemistry

The methods employed to calculate the thermochemical parameters of species are identical to those used in our previous studies,^4,5^ with 0 K formation enthalpies determined via the isodesmic approach using the most recent ATcT values for the molecular and radical chaperones, and uncertainties computed using methods described by Simmie et al.^45^ Temperature-dependent enthalpies, entropies, and heat capacities were calculated using traditional statistical thermodynamic methods as implemented in MESSPF,^42^ with Chemkin format polynomials fitted using PAC99,^46^ and are provided as Supporting Information (SI).

### 2.3 Transition State Theory (TST), Rice–Ramsperger–Kassel–Marcus (RRKM), and Master Equation (ME) Calculations

High-pressure limiting and pressure-dependent rate constants were calculated for the C_2_–C_4_ PESs using RRKM/ME as implemented in MESS,^42^ in which tunneling is accounted for via an asymmetric Eckart model.^47^ To model collisional energy transfer, a single exponential down model was used and is estimated to be ⟨Δ*E*_down_(*T*)⟩ = 75 × (*T*/300)^1.05^ cm^–1^ for the Ċ_2_H_5_ PES^17^ and ⟨Δ*E*_down_(*T*)⟩ = 200 × (*T*/300)^0.75^ cm^–1^ for the Ċ_3_H_7_ and Ċ_4_H_9_ PESs.^48−51^

## 3 Theoretical Results

### 3.1 Thermochemistry

Table 2 presents formation enthalpies, along with their 2σ uncertainties computed via isodesmic and atomization methods. Also presented are ATcT,^52,53^ ANL0,^54^ and ANL1^54^ formation enthalpies with 2σ uncertainties. The current study uses the most recent ATcT values for the molecular and radical chaperones.^52,53^ Similar to previous work,^4,5^ ATcT, ANL0, and ANL1 formation enthalpies do not exist for the species Ċ_4_H_7_-11, Ċ_4_H_7_-12, Ċ_4_H_7_-13, Ċ_4_H_7_-14, Ċ_4_H_7_-22, iĊ_4_H_7_, and iĊ_4_H_7_-i1. Quantum chemical composite methods (CBS–QB3, CBS–APNO, G3, and G4)^55−57^ were therefore used to calculate their formation enthalpies at 0 K via isodesmic reactions suitable for each species, using ATcT values as chaperones.

**Table 2:** Formation Enthalpies and Uncertainties (2σ) Computed via Isodesmic and Atomization Methods, together with ATcT, ANL0, and ANL1 Formation Enthalpies and Uncertainties

species	isodesmic (0 K, kJ mol^–1^)	isodesmic (2σ)	atomization (0 K, kJ mol^–1^)	atomization (2σ)	ATcT^52,53^ (0 K, kJ mol^–1^)	ANL0^54^	ANL1^54^	Burcat^58^ (0 K, kJ mol^–1^)
C_2_H_4_	60.60	0.45	61.36	3.85	60.88	60.20	60.20	61.03
Ċ_2_H_5_	131.65	0.74	131.06	6.65	131.06	131.30	131.00	130.77
Ċ_2_H_3_	301.49	0.96	301.26	5.41	301.13	300.90	300.50	300.87
C_3_H_6_	35.03	0.36	35.85	7.33	34.93	34.50		35.01
nĊ_3_H_7_	117.78	0.66	118.15	9.75	118.34	118.20		119.15
iĊ_3_H_7_	105.33	0.92	105.71	9.63	105.32	105.10		108.24
Ċ_3_H_5_-s	277.86	0.87	278.38	7.53	278.22	278.40		276.29
Ċ_3_H_5_-t	262.28	0.95	262.80	6.86	262.98	263.00		
Ċ_3_H_5_-a	177.44	2.00	179.03	6.69	180.03	179.60		180.40
C_4_H_8_-1	21.15	0.20	22.40	10.66	21.00	21.30		20.82
C_4_H_8_-2	9.40	0.25	10.63	10.86	9.38	9.60		9.39
Ċ_4_H_9_-1	102.20	0.77	102.52	13.08	102.74	103.20		105.91
Ċ_4_H_9_-2	90.76	0.74	91.55	12.82	90.84	90.90		94.95
Ċ_4_H_7_-11	263.61	0.79	263.97	10.27				262.76
Ċ_4_H_7_-12	248.88	0.81	249.16	9.84				248.45
Ċ_4_H_7_-13	152.70	0.81	152.04	9.48				153.55
Ċ_4_H_7_-14	222.83	0.77	224.34	12.26				220.92
Ċ_4_H_7_-22	239.46	1.24	240.21	9.97				239.74
iC_4_H_8_	3.61	0.31	5.19	10.72	4.01	4.20		3.46
iĊ_4_H_9_	96.14	0.73	96.38	12.91	97.17			97.92
tĊ_4_H_9_	73.86	0.73	75.31	13.23	75.60			79.72
iĊ_4_H_7_	153.25	0.81	153.73	9.49				155.27
iĊ_4_H_7_-i1	250.60	0.70	251.18	10.45				

Excellent agreement is observed between this work and the ATcT^52,53^ values, with differences, expressed as mean absolute error (MAE ± 2σ), being on average 0.59 ± 1.38 kJ mol^–1^. Differences between this work and ANL0^54^ and ANL1^54^ computations are on average 0.57 ± 1.03 and 0.68 ± 0.60 kJ mol^–1^, respectively. Differences between this work and Burcat^58^ are slightly higher at 1.58 ± 3.2 kJ mol^–1^. Comparisons between isodesmic and atomization values calculated in the current work are in excellent agreement, with an MAE of 0.76 ± 0.93 kJ mol^–1^. As discussed in our previous work,^4,5^ although the isodesmic and atomization methods give similar nominal 0 K heats of formation, the isodesmic method is often used to achieve “chemical accuracy”. Our computed final heat of formation uncertainties for the isodesmic reactions are between 0.36 and 2.00 kJ mol^–1^.

Table 3 presents 298 K formation enthalpies between this work and literature data, with the results generally in good agreement. Differences between this work and Goldsmith^43^ are on average 1.56 ± 2.61 kJ mol^–1^. Excellent agreement is observed between this work and ATcT, with an MAE of 0.76 ± 1.43 kJ mol^–1^. The values reported by Burcat^58^ are within 2.01 ± 5.90 kJ mol^–1^ of this work.

**Table 3:** Comparisons of the Formation Enthalpies Computed in This Work with Literature Data

species	Δ_f_*H*_298K_ (this work)	Δ_f_*H*_298K_ (Goldsmith)^43^	Δ_f_*H*_298K_ (ATcT)^52,53^	Δ_f_*H*_298K_ (Burcat)^58^
C_2_H_4_	51.99	52.30	52.36	52.50
Ċ_2_H_5_	120.61	120.92	119.99	119.70
Ċ_2_H_3_	297.29	297.90	296.93	296.58
C_3_H_6_	19.88	19.25	19.93	20.00
nĊ_3_H_7_	100.23	101.67	100.94	101.32
iĊ_3_H_7_	87.92	88.70	88.45	90.19
Ċ_3_H_5_-s	267.07	268.19	267.38	265.53
Ċ_3_H_5_-t	251.79	253.13	252.58	237.65
Ċ_3_H_5_-a	165.55	169.87	168.31	168.60
C_4_H_8_-1	–0.21	–0.00	0.05	–0.03
C_4_H_8_-2	–11.30	–11.30	–11.18	–11.19
Ċ_4_H_9_-1	78.86	80.75	80.23	81.80
Ċ_4_H_9_-2	68.02	69.45	66.07	70.22
Ċ_4_H_7_-11	246.82	248.11		245.87
Ċ_4_H_7_-12	232.66			231.16
Ċ_4_H_7_-13	135.21	137.65		136.11
Ċ_4_H_7_-14	206.50	208.36		204.60
Ċ_4_H_7_-22	223.32	225.10		223.85
iC_4_H_8_	–17.60	–17.15	–17.05	–17.57
iĊ_4_H_9_	72.29	74.48	73.18	73.79
tĊ_4_H_9_	50.77	54.39	50.30	55.04
iĊ_4_H_7_	134.68	139.32		137.60
iĊ_4_H_7_-i1	233.84			

Table 4 presents comparisons of entropies calculated in this work and the literature, with differences being larger than those observed for the enthalpies. Differences between Goldsmith^43^ and this work are on average 1.13 ± 3.72 J K^–1^ mol^–1^, while differences between those recommended by Burcat^58^ and calculated here are on average 5.09 ± 11.64 J K^–1^ mol^–1^. In the case of iĊ_3_H_7_, the lowest-energy conformer has *Cs* symmetry, with an assigned symmetry factor of 1. If it is assumed that the symmetry factor of iĊ_3_H_7_ is 2, the entropy value drops from 295.05 to 289.29 J K^–1^ mol^–1^, which is now only 1.01 J K^–1^ mol^–1^ larger than the value computed by Goldsmith and 0.82 J K^–1^ mol^–1^ lower than that by Burcat.^58^ For the Ċ_4_H_7_-14 radical, our computed entropy is 5.91 and 4.45 J K^–1^ mol^–1^ larger than Goldsmith^43^ and Burcat,^58^ respectively. However, Goldsmith^43^ reports an uncertainty of 5.86 J K^–1^ mol^–1^ for their reported entropy for Ċ_4_H_7_-14, and our value falls within this range.

**Table 4:** Comparisons of Entropies Computed in This Work with Literature Data

species	*S*_298K_ (this work)	*S*_298K_ (Goldsmith)^43^	*S*_298K_ (Burcat)^58^
C_2_H_4_	218.66	218.82	219.32
Ċ_2_H_5_	247.38	247.27	242.98
Ċ_2_H_3_	233.38	233.47	233.66
C_3_H_6_	266.10	266.10	266.66
nĊ_3_H_7_	289.91	289.95	290.46
iĊ_3_H_7_	295.05	288.28	290.11
Ċ_3_H_5_-s	271.27	271.54	271.31
Ċ_3_H_5_-t	273.48	273.63	266.06
Ċ_3_H_5_-a	257.07	257.32	257.88
C_4_H_8_-1	307.77	306.27	305.37
C_4_H_8_-2	295.67	295.81	296.33
Ċ_4_H_9_-1	331.26	328.44	307.63
Ċ_4_H_9_-2	331.85	330.54	327.42
Ċ_4_H_7_-11	312.91	311.71	311.28
Ċ_4_H_7_-12	315.08		300.37
Ċ_4_H_7_-13	300.56	301.25	306.09
Ċ_4_H_7_-14	321.80	315.89	317.35
Ċ_4_H_7_-22	310.77	311.28	313.26
iC_4_H_8_	293.21	293.72	287.45
iĊ_4_H_9_	319.07	319.66	304.66
tĊ_4_H_9_	318.97	318.82	323.39
iĊ_4_H_7_	293.08	293.72	300.80
iĊ_4_H_7_-i1	305.54		

Table 5 presents heat capacities for the C_2_–C_4_ species calculated in this work, by Goldsmith^43^ and present in the Burcat database.^58^ A good agreement is observed, with an MAE of 1.69 ± 1.5 J mol^–1^ K^–1^ observed between this work and Goldsmith.^43^ Differences between this work and the Burcat database^58^ are slightly higher, with an MAE of 1.87 ± 3.36 J K^–1^ mol^–1^.

**Table 5:** Comparisons of Heat Capacities Computed Here with Literature Data

	*C*_p_							
species	study	300	400	500	600	800	1000	1500
C_2_H_4_	this work	42.04	51.22	60.64	69.19	82.07	92.25	108.54
	Goldsmith^43^	42.68	52.30	61.50	69.87	82.84	92.88	109.20
	Burcat^58^	43.05	52.64	62.27	70.93	83.89	94.09	109.58
Ċ_2_H_5_	this work	50.83	60.85	70.92	80.13	94.66	106.31	125.31
	Goldsmith^43^	51.46	61.92	71.96	80.75	95.40	107.11	125.94
	Burcat^58^	50.86	61.26	71.64	81.13	96.05	107.91	126.21
Ċ_2_H_3_	this work	43.02	50.42	57.32	63.28	72.12	79.19	90.65
	Goldsmith^43^	43.51	51.46	58.16	63.60	72.80	79.50	91.21
	Burcat^58^	42.20	49.42	56.30	62.33	71.37	78.58	89.98
C_3_H_6_	this work	62.80	77.74	92.34	105.50	125.94	141.81	166.99
	Goldsmith^43^	64.43	79.91	94.56	107.11	127.61	143.09	168.20
	Burcat^58^	64.71	80.19	95.03	108.28	128.79	144.61	168.44
nĊ_3_H_7_	this work	71.53	88.04	103.86	117.96	139.72	156.80	184.17
	Goldsmith^43^	72.38	89.96	105.86	119.24	141.42	158.16	185.35
	Burcat^58^	71.61	88.44	104.39	118.52	140.27	157.27	183.71
iĊ_3_H_7_	this work	67.68	83.16	99.11	113.83	136.88	154.91	183.44
	Goldsmith^43^	68.62	84.94	100.83	115.06	138.49	156.06	184.51
	Burcat^58^	65.81	81.67	97.76	112.60	136.04	154.33	182.33
Ċ_3_H_5_-s	this work	62.51	75.45	87.65	98.44	115.11	128.06	148.67
	Goldsmith^43^	64.02	77.40	89.54	99.58	116.32	129.29	149.37
	Burcat^58^	63.63	76.53	88.46	98.97	115.47	128.30	148.22
Ċ_3_H_5_-t	this work	61.98	74.30	86.40	97.33	114.35	127.61	148.57
	Goldsmith^43^	63.18	76.15	87.86	98.74	115.90	128.87	149.37
	Burcat^58^	61.94	76.98	90.79	102.80	121.04	134.95	155.57
Ċ_3_H_5_-a	this work	61.33	76.91	90.74	102.38	119.28	132.16	152.54
	Goldsmith^43^	62.34	78.24	92.05	102.93	120.08	133.05	153.13
	Burcat^58^	62.12	77.74	91.51	103.04	119.77	132.52	152.17
C_4_H_8_-1	this work	84.31	105.66	125.90	143.81	171.31	192.50	225.95
	Goldsmith^43^	87.03	109.20	129.29	146.44	173.64	194.56	227.61
	Burcat^58^	85.96	106.28	126.08	144.16	173.16	195.04	227.47
C_4_H_8_-2	this work	85.63	105.06	124.29	141.89	169.82	191.32	225.43
	Goldsmith^43^	88.28	108.78	127.61	144.77	172.38	193.30	226.77
	Burcat^58^	88.03	108.22	127.84	145.62	173.80	195.38	227.77
Ċ_4_H_9_-1	this work	93.56	116.39	137.92	156.88	185.81	208.19	243.65
	Goldsmith^43^	96.23	119.24	140.58	158.57	187.86	210.04	245.18
	Burcat^58^	94.98	118.67	140.97	160.63	190.76	213.94	249.44
Ċ_4_H_9_-2	this work	90.04	111.14	132.42	151.82	181.88	205.36	242.30
	Goldsmith^43^	91.63	113.80	135.14	153.97	184.10	207.53	243.93
	Burcat^58^	86.79	109.43	131.47	151.27	181.88	205.47	241.32
Ċ_4_H_7_-11	this work	84.17	103.50	121.34	136.89	160.62	178.88	207.72
	Goldsmith^43^	86.19	106.27	123.85	138.91	162.34	180.33	208.78
	Burcat^58^	84.05	103.05	120.71	136.20	160.05	178.45	206.63
Ċ_4_H_7_-12	this work	83.70	102.27	120.04	135.77	159.75	178.27	207.41
	Goldsmith^43^							
	Burcat^58^	84.33	103.88	121.97	137.87	162.53	181.46	210.28
Ċ_4_H_7_-13	this work	81.56	101.65	120.62	137.24	162.13	181.10	210.74
	Goldsmith^43^	83.26	103.76	122.59	138.49	163.59	182.42	211.71
	Burcat^58^	81.15	101.15	120.07	136.69	161.70	180.78	209.69
Ċ_4_H_7_-14	this work	83.27	102.21	120.16	135.94	159.76	178.10	207.05
	Goldsmith^43^	86.61	105.86	123.43	138.49	161.92	179.91	208.36
	Burcat^58^	85.14	104.57	122.89	138.96	163.17	181.76	210.14
Ċ_4_H_7_-22	this work	83.12	99.93	116.93	132.58	157.36	176.52	206.64
	Goldsmith^43^	84.94	102.81	119.24	134.31	158.41	178.24	207.94
	Burcat^58^	83.51	99.85	116.58	132.13	157.01	176.30	205.65
iC_4_H_8_	this work	86.01	106.46	125.84	143.20	170.72	191.87	225.62
	Goldsmith^43^	88.28	109.20	128.87	145.60	172.80	193.72	227.19
	Burcat^58^	86.44	109.53	130.81	149.22	176.71	197.59	228.66
iĊ_4_H_9_	this work	95.21	118.37	139.73	158.34	186.70	208.68	243.73
	Goldsmith^43^	96.65	120.92	142.26	160.25	188.70	210.46	245.18
	Burcat^58^	98.56	122.36	143.90	162.52	191.01	212.96	246.99
tĊ_4_H_9_	this work	88.45	108.45	129.49	149.13	180.04	204.10	241.76
	Goldsmith^43^	90.79	111.29	132.21	151.04	182.00	205.85	243.09
	Burcat^58^	82.78	104.42	126.31	146.47	178.31	202.90	240.05
iĊ_4_H_7_	this work	80.50	102.35	121.72	138.17	162.72	181.35	210.69
	Goldsmith^43^	82.01	104.18	123.43	139.33	164.01	182.42	211.71
	Burcat^58^	82.59	103.51	122.32	138.44	162.74	181.30	209.76
iĊ_4_H_7_-i1	this work	85.77	103.91	120.75	135.71	159.46	177.78	207.03
	Goldsmith^43^							
	Burcat^58^							

### 3.2 Reactions of Ḣ atoms with C_2_H_4_, C_3_H_6_, C_4_H_8_-1, C_4_H_8_-2, and iC_4_H_8_

**Figure 1:**
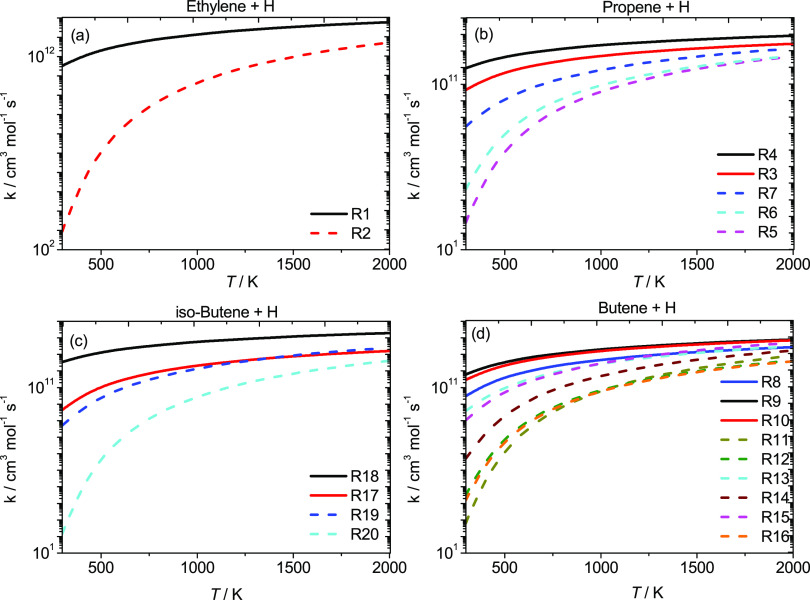
High-pressure limiting rate constants for the reactions of (a) ethylene + Ḣ, (b) propene + Ḣ, (c) isobutene + Ḣ, and (d) 1- and 2-butene + Ḣ.

Figure 1 compares the high-pressure limiting rate constants (Table 6) for (a) ethylene + Ḣ, (b) propene + Ḣ, (c) isobutene + Ḣ, and (d) 1- and 2-butene + Ḣ. Hydrogen atom addition to, and abstraction from, ethylene have computed energy barriers of 11.2 and 63.1 kJ mol^–1^, respectively. Terminal Ḣ atom addition to propene has a computed energy barrier of 8.4 kJ mol^–1^, which is 7.2 kJ mol^–1^ lower than that for internal addition. As expected, Ḣ atom abstraction from the primary allylic site of propene is favored, with an energy barrier of 31.1 kJ mol^–1^. Abstraction of the two Ḣ atoms on the primary vinylic site have similar barriers of 63.7 and 64.6 kJ mol^–1^, leading to cis- and trans-configurations of Ċ_3_H_5_-s, respectively. Terminal Ḣ atom addition to isobutene forming the tertiary tĊ_4_H_9_ radical has a computed barrier of 6.1 kJ mol^–1^, which is 15.0 kJ mol^–1^ lower than internal addition forming the primary iĊ_4_H_9_ radical. Abstraction from the primary allylic site has a computed barrier of 30.5 kJ mol^–1^. Terminal and internal Ḣ atom addition can exist for 1-butene, with respective barriers of 9.3 and 16.0 kJ mol^–1^, while abstraction from the primary allylic site has a barrier of 22.85 kJ mol^–1^. Internal addition to 2-butene and abstraction from the primary allylic site have respective barriers of 12.5 and 33.5 kJ mol^–1^.

**Table 6:** Computed Energy Barriers, Heats of Reaction, and High-Pressure Limiting Rate Constant (298–2000 K) for the Reactions of Ḣ Atoms with C_2_–C_4_ Alkenes^a^

		reaction	Δ^⧧^*H*_0K_	Δ_r_*H*_0K_	*A*	*n*	*E*_a_
C_2_	R1	C_2_H_4_ + Ḣ ↔ Ċ_2_H_5_	11.18	–146.48	1.15 × 10^15^	–0.41	14.73
	R2	C_2_H_4_ + Ḣ ↔ Ċ_2_H_3_ + H_2_	63.12	24.09	4.79 × 10^05^	2.55	51.77
C_3_	R3	C_3_H_6_ + Ḣ ↔ nĊ_3_H_7_	15.61	–132.97	6.25 × 10^15^	–0.73	19.34
	R4	C_3_H_6_ + Ḣ ↔ iĊ_3_H_7_	8.39	–146.83	1.02 × 10^14^	–0.03	11.43
	R5	C_3_H_6_ + Ḣ ↔ Ċ_3_H_5_-s + H_2_	63.97	27.14	1.21 × 10^06^	2.43	53.96
	R6	C_3_H_6_ + Ḣ ↔ Ċ_3_H_5_-t + H_2_	51.95	12.04	3.11 × 10^05^	2.51	40.36
	R7	C_3_H_6_ + Ḣ ↔ Ċ_3_H_5_-a + H_2_	31.09	–71.88	6.97 × 10^02^	3.24	13.93
C_4_	R8	C_4_H_8_-1 + Ḣ ↔ Ċ_4_H_9_-1	16.04	–136.49	2.23 × 10^14^	–0.27	18.47
	R9	C_4_H_8_-1 + Ḣ ↔ Ċ_4_H_9_-2	9.32	–147.65	6.06 × 10^15^	–0.60	14.59
	R10	C_4_H_8_-2 + Ḣ ↔ Ċ_4_H_9_-2	12.52	–136.08	1.56 × 10^15^	–0.42	15.68
	R11	C_4_H_8_-1 + Ḣ ↔ Ċ_4_H_7_-11 + H_2_	64.81	27.38	2.01 × 10^06^	2.44	54.53
	R12	C_4_H_8_-1 + Ḣ ↔ Ċ_4_H_7_-12 + H_2_	52.29	13.06	2.11 × 10^05^	2.54	40.67
	R13	C_4_H_8_-1 + Ḣ ↔ Ċ_4_H_7_-13 + H_2_	22.85	–82.85	2.37 × 10^05^	2.56	12.24
	R14	C_4_H_8_-1 + Ḣ ↔ Ċ_4_H_7_-14 + H_2_	42.70	–14.76	1.23 × 10^05^	2.71	29.03
	R15	C_4_H_8_-2 + Ḣ ↔ Ċ_4_H_7_-13 + H_2_	33.51	–69.77	2.60 × 10^04^	2.95	15.36
	R16	C_4_H_8_-2 + Ḣ ↔ Ċ_4_H_7_-22 + H_2_	54.25	15.91	1.21 × 10^04^	2.41	43.19
	R17	iC_4_H_8_ + Ḣ ↔ iĊ_4_H_9_	21.15	–124.43	9.67 × 10^13^	–0.21	22.08
	R18	iC_4_H_8_ + Ḣ ↔ tĊ_4_H_9_	6.13	–145.60	7.89 × 10^15^	–0.53	11.98
	R19	iC_4_H_8_ + Ḣ ↔ iĊ_4_H_7_ + H_2_	30.50	–63.73	4.45 × 10^03^	3.08	14.81
	R20	iC_4_H_8_ + Ḣ ↔ iĊ_4_H_7_-i1 + H_2_	66.75	32.19	2.60 × 10^06^	2.34	57.34

a Units (*AT^n^* = cm^3^ mol^–1^ s^–1^, energies kJ mol^–1^).

**Figure 2:**
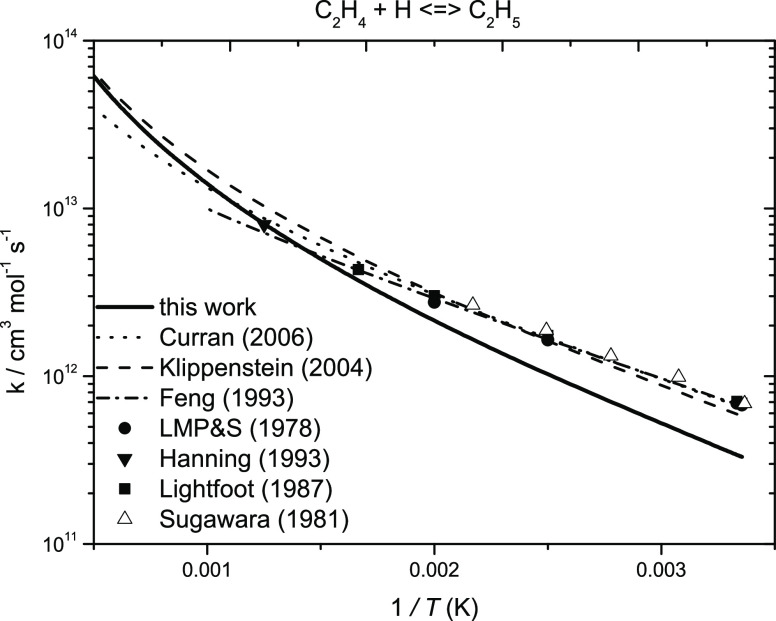
High-pressure limiting rate constant comparisons for the reactions of Ḣ atom addition with ethylene. The solid line represents the current work (ROCCSD(T)/aug-cc-pVXZ); dotted line, Curran;^59^ dashed line, Miller and Klippenstein;^17^ dashed-dotted line, Feng et al.^7^ and Lee et al.;^9^ ▲, Hanning-Lee et al.;^8^ ■, Lightfoot et al.;^10^ and △, Sugawara et al.^14^

Figure 2 compares theoretical and experimental data^8−10,14^ for the reaction C_2_H_4_ + Ḣ ↔ Ċ_2_H_5_. Also plotted is the rate constant recommendation from Curran et al.^59^ and the transition state theory fit to the experiments by Feng et al.,^7^ with a good agreement being observed. The largest difference observed between the current work and Miller and Klippenstein^17^ is a factor of 1.75 at 300 K. The difference in energy barrier of 0.54 kJ mol^–1^ and the quoted uncertainty of their fits to replicate the master equation results of ±20%, which would account for an accumulative difference of ∼1.5.

**Figure 3:**
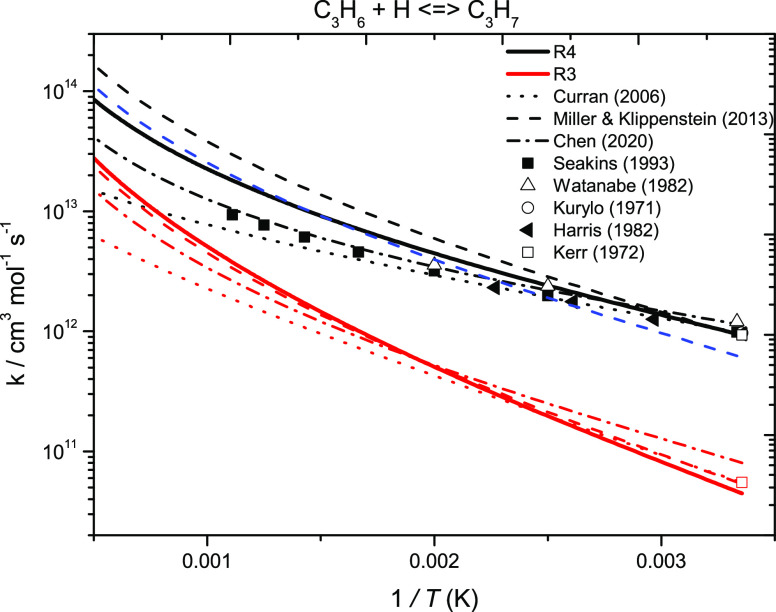
High-pressure limiting rate constant comparisons for the reactions of Ḣ atom addition to propene. Solid lines represent the current work (ROCCSD(T)/aug-cc-pVXZ); dotted lines, Curran;^59^ dashed lines, Miller and Klippenstein;^18^ dashed-dotted lines, Chen;^20^ ■, Seakins et al.;^23^ △, Watanabe et al.;^27^ ○, Kurylo et al.;^26^ ◀, Harris et al.;^31^ and □, Kerr et al.^24^

Figure 3 presents high-pressure limiting rate constant comparisons for the Ḣ atom addition reactions to propene. A relatively good agreement is observed between the current work and theory and experiments from the literature. To improve agreement with experiment, Miller and Klippenstein^18^ altered some reaction barriers, including those for terminal and internal H-atom addition and H-atom abstraction from the primary allylic site of propene. The adjusted rate constant for internal addition to propene (red) is in excellent agreement with the one calculated in the current work, and the adjusted energy barrier of 15.5 kJ mol^–1^ is almost identical to 15.6 kJ mol^–1^ calculated in the current work, as shown in Table 6. The rate constant for terminal addition (black) is approximately a factor of 2 faster than that calculated here. However, as mentioned by Chen et al.,^20^ the higher values reported by Miller and Klippenstein may be attributed to input data errors. An error in symmetry number affects the energy barriers and pressure-dependent rate constant expressions. If the effect of symmetry reduced the rate constant by a factor of ∼1.5 (dashed blue line, Figure 3), it would be in good agreement with that calculated here.

The rate constants reported by Chen et al.^20^ are within a factor of 2 of the current work over the temperature range 298–2000 K. Differences in energy barriers computed in this work and that by Chen are 3.01 and 2.49 kJ mol^–1^ for nonterminal addition and terminal addition, respectively. The recommendations by Curran et al.^59^ are in good agreement at *T* < 800 K, but differences become larger at higher temperatures, with a factor of ∼5 discrepancy observed at 2000 K.

**Figure 4:**
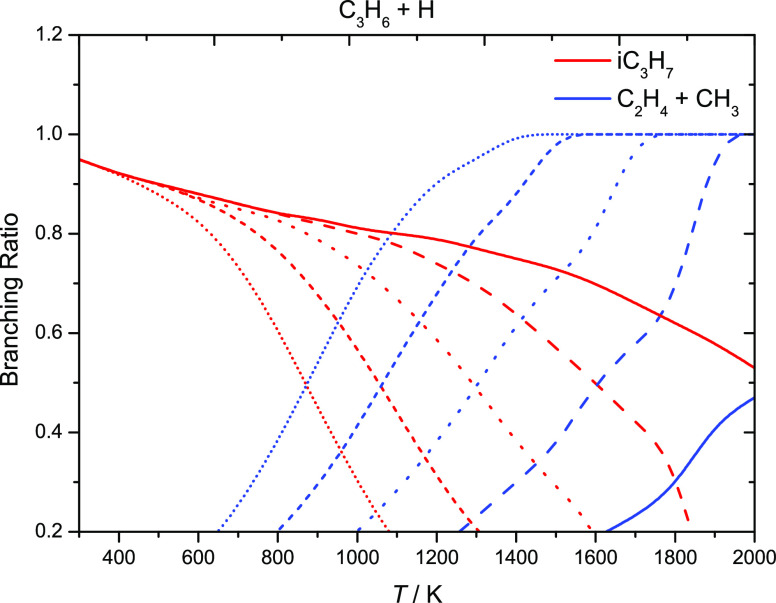
Temperature- and pressure-dependent branching ratios for propene + Ḣ via hydrogen atom addition reactions at 0.1 (short-dotted lines), 1 (short-dashed lines), 10 (dotted lines), 100 (dashed lines), and 1000 (solid lines) atm.

Figure 4 presents the temperature and pressure dependencies of the product branching ratios for Ḣ atom addition to propene in the temperature range 298–2000 K and at pressures of 0.1, 1.0, 10, 100, and 1000 atm. At 0.1 atm, Ḣ atom addition to propene forming iĊ_3_H_7_ radicals is favored at temperatures up to 800 K, until the formation of C_2_H_4_ and ĊH_3_ dominates. For pressures of 1.0, 10, and 100 atm, the formation of iĊ_3_H_7_ is favored at temperatures up to ∼1000, 1200, and 1500 K, respectively.

**Figure 5:**
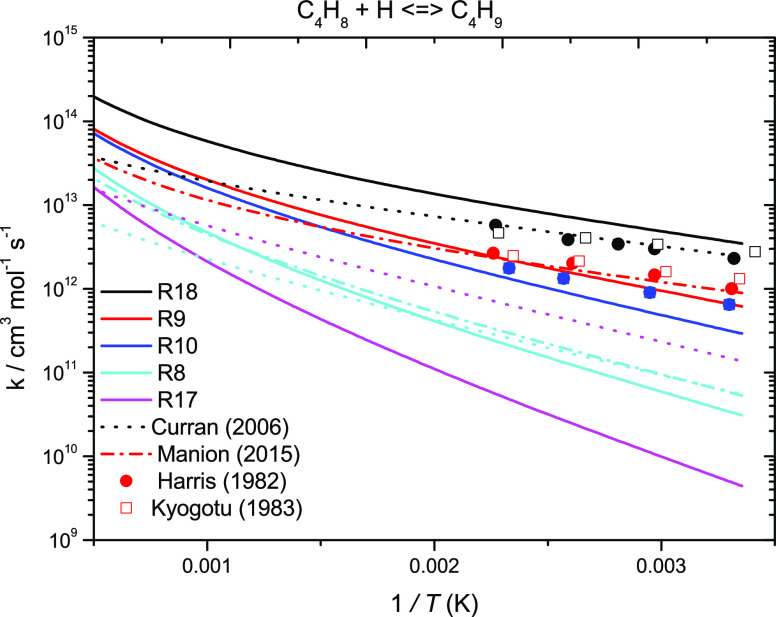
High-pressure limiting rate constant comparisons for the reactions of Ḣ atom addition to the butene isomers. Solid lines represent the current work (ROCCSD(T)/aug-cc-pVXZ); dotted lines, Curran;^59^ dash-dotted lines, Manion et al.;^19^ red color solid circle, Harris et al.;^31^ and red color square, Kyogotu et al.^60^

Figure 5 presents high-pressure limiting rate constant comparisons for the reactions of Ḣ atom addition to the butene isomers. Larger differences are observed for the reactions of Ḣ atoms with C_4_ alkenes calculated here and in the literature. For terminal addition to 1-butene, the rate constants determined by Manion et al.^19^ and in this work are within a factor of ∼2.22 over the temperature range 298–2000 K. The rate constants for internal addition to 1-butene are in excellent agreement and are within a factor of ∼1.3. Additionally, the current calculations are in relatively good agreement with the experimental data by Kyogutu et al.^60^ and Harris et al.^31^ For terminal addition to isobutene, the recommendations by Curran et al.^59^ are again in good agreement at lower temperatures, but there is a larger deviation of a factor of 5 observed at 2000 K. The largest difference is observed for internal addition to isobutene. However, the difference in rate constants calculated in the current work for internal addition to 1-butene and isobutene is consistent with the difference in the computed barrier heights of 5.1 kJ mol^–1^, accounting for the factor of 7 discrepancy at low temperatures. Curran’s recommendation is a factor of ∼30 times faster at 298 K. The rate constant recommendation used is 2.5 times the recommendation used for internal addition to propene. However, it was found that our calculation for internal addition to propene is ∼10 times faster than that to isobutene at 298 K, which can be attributed to the energy barrier for internal addition to propene being ∼5.54 kJ mol^–1^ lower than that for isobutene.

**Figure 6:**
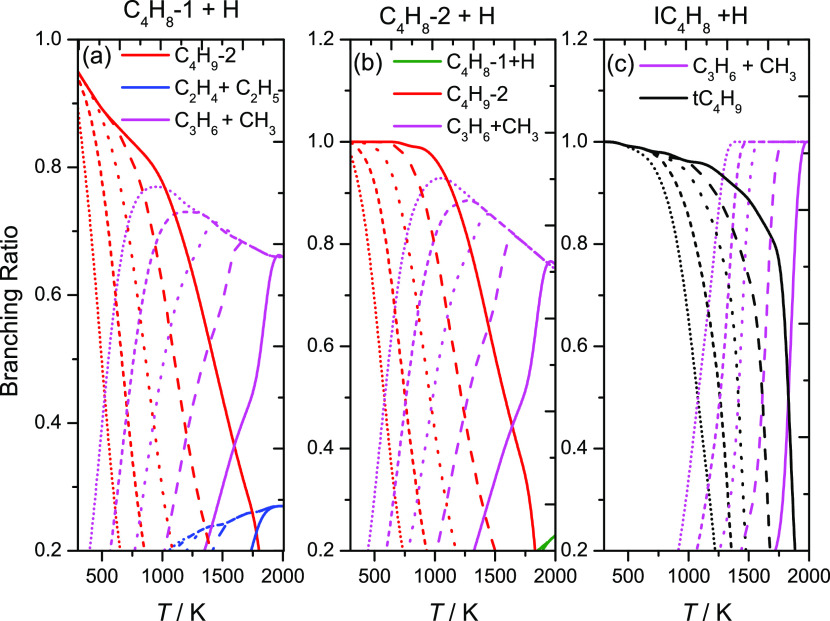
Temperature- and pressure-dependent branching ratios for (a) 1-butene, (b) 2-butene, and (c) isobutene via hydrogen atom addition reactions at 0.1 (short-dotted lines), 1 (short-dashed lines), 10 (dotted lines), 100 (dashed lines), and 1000 (solid lines) atm.

Figure 6 shows the temperature and pressure dependencies of the product branching ratios for Ḣ atom addition to (a) 1-butene, (b) 2-butene, and (c) isobutene in the temperature range 298–2000 K and at pressures of 0.1, 1.0, 10, 100, and 1000 atm. For both 1- and 2-butene, at 0.1 atm, Ḣ atom addition forming the Ċ_4_H_9_-2 radical is favored at temperatures up to 500 K. The formation of C_3_H_6_ and ĊH_3_ then dominates the reaction flux at higher temperatures. Similar trends are observed in Figure 6a,b at 1.0, 10, and 100 atm. However, the formation of Ċ_4_H_9_-2 is favored at temperatures up to ∼700, 900, and 1200 K, respectively. In the case of isobutene, Ḣ atom addition forming tĊ_4_H_9_ radicals is favored at temperatures up to 1000 K at 0.1 atm, whereas at higher temperatures, the formation of C_3_H_6_ and ĊH_3_ dominates. The same trends are observed at 1.0, 10, and 100 atm. However, the formation of tĊ_4_H_9_ radicals is favored at temperatures up to 1200, 1400, and 1600 K, respectively.

**Figure 7:**
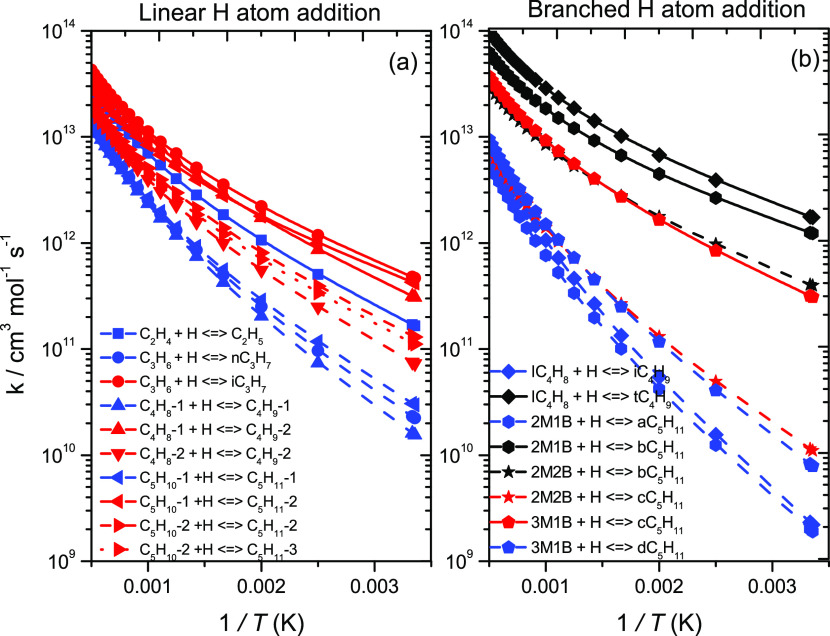
Rate constants (symmetry-uncorrected) for terminal and internal Ḣ atom addition to (a) linear and (b) branched C_2_–C_5_ alkenes from previous^4,5^ and current works. Solid and dashed lines represent terminal and internal addition, respectively. Different colors represent different radical types formed. Black (tertiary), red (secondary), and blue (primary). Different symbols correspond to the different reactants. Blue box solid (ethylene), blue circle solid (propene), red triangle up solid (1-butene), red triangle down solid (2-butene), black diamond solid (isobutene), red triangle left (1-pentene), red triangle right (2-pentene), hexagon solid (2-methyl-1-butene), black star solid (2-methyl-2-butene), and blue pentagon open solid (3-methyl-1-butene).

Figure 7 presents rate constants for Ḣ atom addition reactions, which are reported with no symmetry or optical isomer corrections between the transition state and reactants—i.e., the reaction path degeneracy is set to 1. Table S1 of Supporting Information presents the symmetry factors for the reactants and transition states prior to this change. As expected, external Ḣ atom addition to each of the alkenes (solid lines) dominates over internal addition (dashed lines). For the linear alkenes, both external and internal Ḣ atom additions can lead to the formation of primary (blue) or secondary radicals (red). The rate constants for external addition to propene, 1-butene, and 1-pentene are similar with respective barrier heights of 8.4, 9.3, and 7.8 kJ mol^–1^. However, the rate constant for external addition to ethylene is approximately a factor of 2 slower than external addition to propene and 1-butene at 500 K, reducing to a factor of ∼1.4 at 2000 K. This difference can be attributed to the difference in energy barrier of ∼2.8 kJ mol^–1^. This can also be correlated with radical stability as a primary radical is formed in the case of ethylene, while secondary radicals are formed for propene, 1-butene, and 1-pentene.

**Figure 8:**
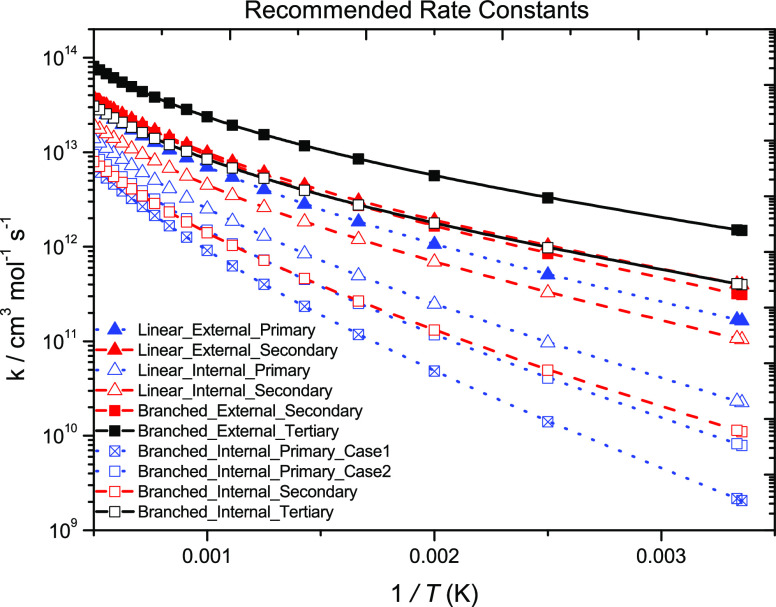
Rate constant recommendations (symmetry-uncorrected) for Ḣ atom addition to linear (▲) and branched (■) C_2_–C_5_ alkenes. Solid, dashed, and dotted lines represent the formation of tertiary, secondary, and primary radicals, respectively. Open symbols are internal C-atom additions; solid symbols are external C-atom additions.

Internal Ḣ atom addition to linear alkenes form either primary (C_3_H_6_ + Ḣ ↔ nĊ_3_H_7_, C_4_H_8_-1 + Ḣ ↔ Ċ_4_H_9_-1, and C_5_H_10_-1 + Ḣ ↔ Ċ_5_H_11_-1) or secondary radicals (C_4_H_8_-2 + Ḣ ↔ Ċ_4_H_9_-2, C_5_H_10_-2 + Ḣ ↔ Ċ_5_H_11_-2, and C_5_H_10_-2 + Ḣ ↔ Ċ_5_H_11_-3). Rate constants for internal addition to propene, 1-butene, and 1-pentene are similar. Rate constants for internal addition to 2-pentene are almost identical, with internal addition to 2-butene being slightly slower. However, this can be attributed to an energy barrier difference of ∼1.6 kJ mol^–1^. The branched alkenes have been described previously,^5^ so we shall not reiterate here. A trend was observed in that the rate constants for the formation of tertiary radicals are the fastest, followed by secondary and primary radicals, respectively.^5^ In the rate rule determinations, two rules were proposed for internal Ḣ-atom addition to branched alkenes (one for addition to a branched alkene where the branching occurs at the double bond and a second for where the branching does not occur at the double bond). For the cases where branching occurs at the double bond (iC_4_H_8_ + Ḣ ↔ iĊ_4_H_9_ and 2M1B + Ḣ ↔ aĊ_5_H_11_), the energy barriers are similar, being 21.15 and 19.9 kJ mol^–1^, and are higher than that for 3M1B + Ḣ ↔ dĊ_5_H_11_ (17.15 kJ mol^–1^), where the branching does not occur at the double bond (Figure 8).

Recommended rate constants were suggested based on (i) whether addition is to a linear or branched alkene, (ii) whether it is terminal or internal addition, and (iii) the type of radical formed. An average of the rate constants within each subclass was taken as the recommended rate constant. If only one rate constant was available, for example, in the case of internal addition to a branched alkene forming a secondary radical (bC_5_H_10_ + Ḣ ↔ cĊ_5_H_11_), the rate constant for the reaction is taken as the recommended rate constant. For the rate constant recommendations presented in Tables 7, 9, and 11, the activation energies are expressed in cal mol^–1^ units for ease in implementing into kinetic mechanisms.

**Table 7:** Rate Constant Recommendations (Symmetry-Uncorrected) for Ḣ Atom Addition to Linear and Branched Alkenes (C_2_–C_5_)

structure	site	radical formed (deg)	*A*	*n*	*E*_a_	uncertainty bounds (upper, lower)
linear	external	1	2.40 × 10^08^	1.60	1526.	
	external	2	4.35 × 10^08^	1.54	1144.	1.17, 1.23
	internal	1	7.79 × 10^07^	1.67	2276.	1.32, 1.45
	internal	2	2.74 × 10^08^	1.52	1621.	1.24, 1.43
branched	external	2	4.21 × 10^08^	1.54	1292.	
	external	3	1.42 × 10^09^	1.47	836.	1.22,1.29
	Internal_Case1	1	2.27 × 10^07^	1.78	3326.	1.18, 1.21
	Internal_Case2	1	4.29 × 10^07^	1.71	2677.	
	internal	2	5.09 × 10^07^	1.65	2401.	
	internal	3	5.45 × 10^08^	1.47	1070.	

**Table 8:** Symmetry Corrections to Be Applied to Rate Constant Recommendations for Ḣ Atom Addition to Alkenes

σ reactant	σ transition state	symmetry-corrected/symmetry-uncorrected
1	0.5	2
2	0.5	4
2	1.0	2
4	2.0	2

In relation to the uncertainty bounds presented in Tables 7, 9, and 11, upper and lower bounds are given, which are defined as
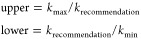
3where *k*_recommendation_ refers to the recommended rate coefficient and *k*_min_ and *k*_max_ refer to the minimum and maximum rate coefficients used in the determinations of the recommended rate coefficients, respectively. Appropriate symmetry corrections must be applied (Table 8) to these recommendations for use in rate rule determinations (Table 9).

**Table 9:** Recommended Rate Constants for H-Atom Abstraction from Alkenes on a Per H-Atom Basis^a^^,^^b^

class	*A*	*n*	*E*_a_	uncertainty bounds (upper, lower)
primary	4.69 × 10^04^	2.68	6959	1.42, 1.35
primary allylic: 1-alkenes	9.14 × 10^02^	3.06	3582	1.10, 1.28
primary allylic: 2-alkenes	1.32 × 10^03^	3.08	3203	1.19, 1.50
primary vinylic	2.72 × 10^05^	2.54	12819	2.39, 2.95
secondary	4.08 × 10^05^	2.44	4734	
secondary allylic	1.06 × 10^05^	2.59	2654	1.67, 2.67
secondary vinylic	2.41 × 10^05^	2.55	9611	2.09, 2.02
tertiary allylic	2.10 × 10^06^	2.19	2329	

a cm^3^ mol^–1^ s^–1^ cal^–1^ units.

b (*AT^n^* = cm^3^ mol^–1^ s^–1^, energies = cal mol^–1^). Fit between 300 and 2000 K.

**Figure 9:**
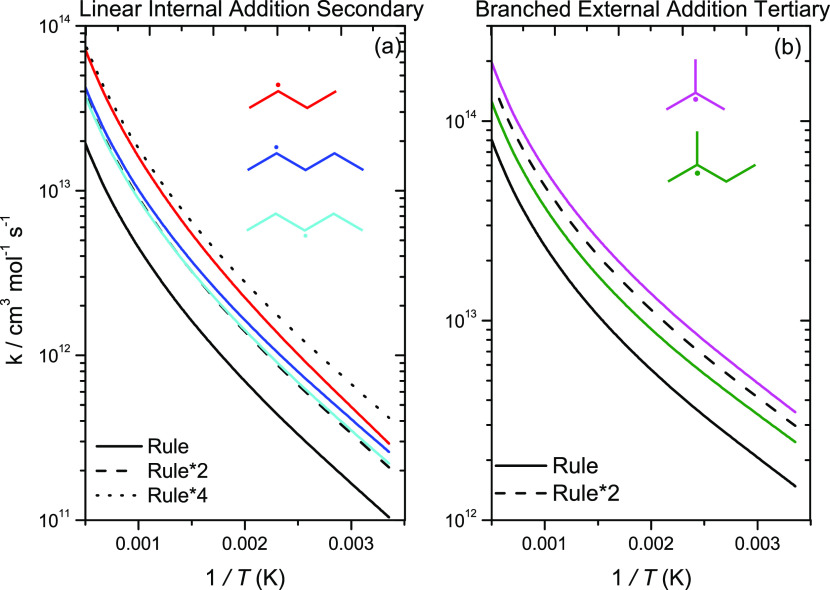
Examples of the application of the proposed rules for Ḣ atom addition to alkenes.

Figure 9 illustrates an example of the rules proposed for (a) internal Ḣ addition to a linear alkene forming a secondary radical and (b) external addition to a branched alkene forming a tertiary radical. The rule is represented by a black solid line. Factors of 2 and 4 variations in the rule are represented by dashed and dotted lines, respectively. The colored lines represent the symmetry-corrected rate constants for each respective reaction, with the uncertainty bounds presented in Table 7. Presented in Figure 9a are the recommended rate constants (symmetry-uncorrected), which is multiplied by 4 for C_4_H_8_-2 + Ḣ ↔ Ċ_4_H_9_-2 since the reactant has a symmetry factor of 2 and the transition state has a symmetry factor of 0.5. For C_5_H_10_-2 + Ḣ ↔ Ċ_5_H_11_-2 and C_5_H_10_-2 + Ḣ ↔ Ċ_5_H_11_-3, the rule is multiplied by 2, since the reactant has a symmetry factor of 1, and both TSs have a symmetry factor of 0.5. As mentioned earlier, Table S1 of Supporting Information presents the symmetry factors for reactants and transition states prior to changing them to 1. It was found that this change decreased each Ḣ atom addition rate constant for both linear and branched alkenes by a factor of 2, with the exception of C_4_H_8_-2 + Ḣ ↔ Ċ_4_H_9_-2, which is explained above.

#### 3.2.1 Branching Ratios of Terminal/Internal Ḣ Atom Addition to Linear and Branched Alkenes

**Figure 10:**
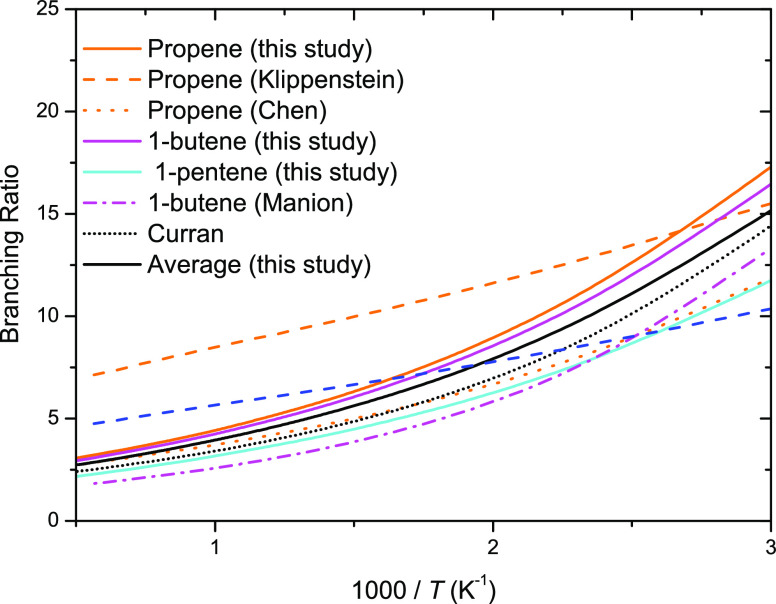
Branching ratio for terminal to internal Ḣ atom addition to (a) linear and (b) branched 1-alkenes.

As discussed earlier, Manion et al.^19^ carried out a shock-tube study to investigate the kinetics of terminal and internal Ḣ atom addition to 1-butene. They observed a factor of 3 discrepancy in the branching ratio for terminal/internal Ḣ atom addition compared to that calculated by Miller and Klippenstein^18^ for the Ḣ + propene reactions. Manion et al.^19^ state that the difference is well outside the experimental error of their experiments or the expected differences for 1-butene. One of the aims of the current work is thus to investigate the branching ratio of terminal to internal Ḣ atom addition in 1-alkenes. Branching ratios for Ḣ atom addition to linear 1-alkenes for C_2_–C_5_ alkenes are plotted in Figure 10. The branching ratios for propene and 1-butene calculated in the current work are within 5% of each other, while our calculated branching ratio for 1-pentene is approximately 40–48% lower than that for propene. Nonterminal addition to pentene is ca. 1.33–1.92 times faster than that for propene and 1-butene at *T* < 300 K. However, terminal addition to propene and 1-butene is 1.2–1.38 times faster than for 1-pentene at *T* > 1000 K. The solid black line represents an average of the calculated rate constants for external addition to a linear 1-alkene forming a secondary radical to internal addition to a linear alkene forming a primary radical and is in excellent agreement with Curran’s recommendation,^59^ with the branching ratios being within 10% of each other. This average branching ratio is also in good agreement with Manion’s branching ratio for 1-butene and is within a factor of 1.57 at 2000 K. The dashed blue line is the branching ratio for terminal/nonterminal addition if the rate constant for terminal addition by Miller and Klippenstein^18^ was reduced by a factor of 1.5. This adjusted branching ratio still differs with that of Manion’s by a factor of ∼2.6 and a factor of 1.5–2.0 of the branching ratios calculated in the current work at 2000 K.

**Figure 11:**
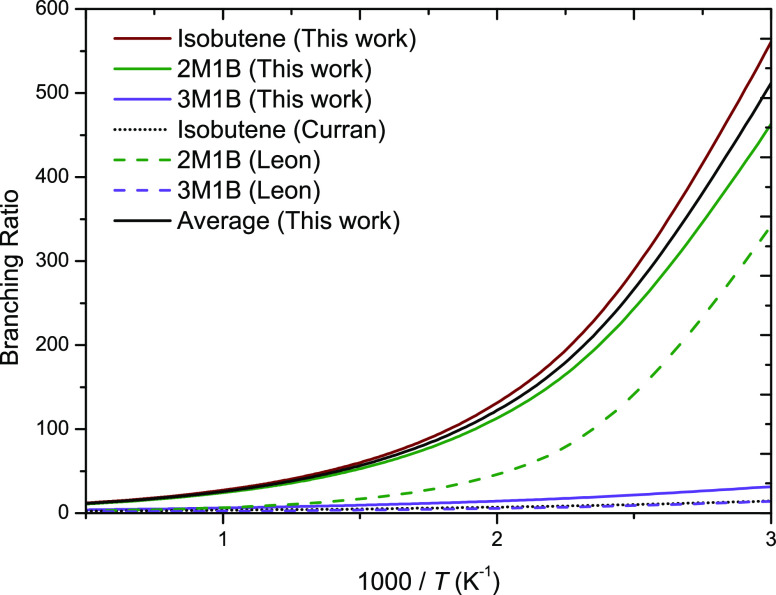
Branching ratio for terminal to internal Ḣ atom addition to branched 1-alkenes.

Figure 11 presents branching ratios for terminal to internal Ḣ atom addition to branched 1-alkenes. 2-Methyl-1-butene (2M1B) and isobutene have branching ratios of 24.2 and 27.2, respectively, at 1000 K. These branching ratios are significantly higher than 3-methyl-1-butene, where the branching ratio of terminal to internal Ḣ atom addition is 6.21 at 1000 K. This is due to branching at the position of the double bond. This results in terminal addition to 2M1B and isobutene forming a tertiary radical, which is more stable than a secondary radical formed through terminal addition to 3M1B, resulting in faster rate constants for terminal addition. Again, the solid black line represents the branching ratio of our recommended rate constants of external addition to branched 1-alkene forming a tertiary radical to internal addition to a branched alkene forming a primary radical. As mentioned earlier, large deviations in rate constants for isobutene are observed between this work and the recommendations by Curran,^59^ particularly for internal Ḣ atom addition. However, Curran does state that no experimental studies for internal Ḣ atom addition existed, so the rate constant recommendation was taken as 2.5 times the rate constant of internal Ḣ atom addition to propene. Manion^19^ states in his study that their^19^ rates should not be applied to 1-olefins that have branching at the double bond position. We also observe that branching at the double bond significantly influences the branching ratio of 1-olefins and explains the difference as why the branching ratio from Curran is lower than that of the current work. Additionally, Manion^19^ states that direct information is lacking on the impact of branching removed from the double bond, but he believes it would have a minimal effect, which is also supported by our calculations here, where our calculated branching ratio for 3M1B is 6.21 at 1000 K. Our calculated branching ratios for propene, 1-butene, and 1-pentene are 4.42, 4.24, and 3.17, respectively, at 1000 K.

**Figure 12:**
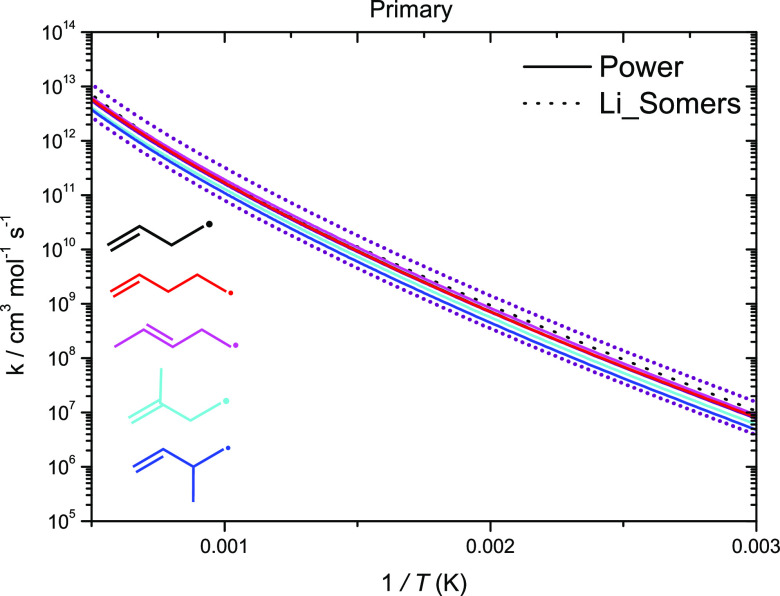
High-pressure limiting rate constants for H-atom abstraction from alkylic (primary) carbon sites on a per Ḣ atom basis.

Rate constant comparisons for the H-atom abstraction reactions from C_2_–C_4_ alkenes were discussed in our previous studies^4,5^ on the reactions of Ḣ atoms with the pentene isomers, so we shall be brief here. As mentioned earlier, excellent agreement is observed for H-atom abstraction from the primary carbon sites (Figure 12). An average of our computed rate constants for abstraction from the primary carbon site as well as the rate calculated by Li et al.^2^ is taken as the recommended rate constant. A good agreement is also observed for the abstraction reactions from the primary allylic and vinylic carbon sites.

**Figure 13:**
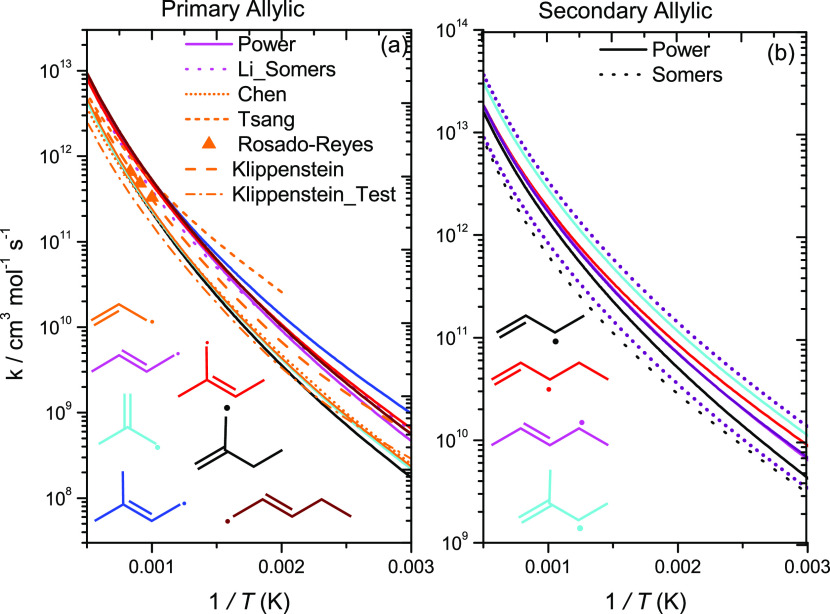
High-pressure limiting rate constants for H-atom abstraction from allylic carbon sites on a per Ḣ atom basis.

For H-atom abstraction from the primary allylic carbon sites, a trend was observed in which abstraction from 2-alkenes is faster than that from 1-alkenes (Figure 13a). As a result, two rate constant recommendations were proposed. The average energy barriers for abstraction from the primary allylic site of 1-alkenes and 2-alkenes computed in this work are 31.0 and 28.6 kJ mol^–1^, respectively, which accounts for most of the difference observed. The difference observed at higher temperatures can be attributed to the difference in entropy of activation. For the rate constant recommendation for 1-alkenes, an average of the rates calculated in this work and previous studies as well as that by Chen et al.^20^ is taken.

**Figure 14:**
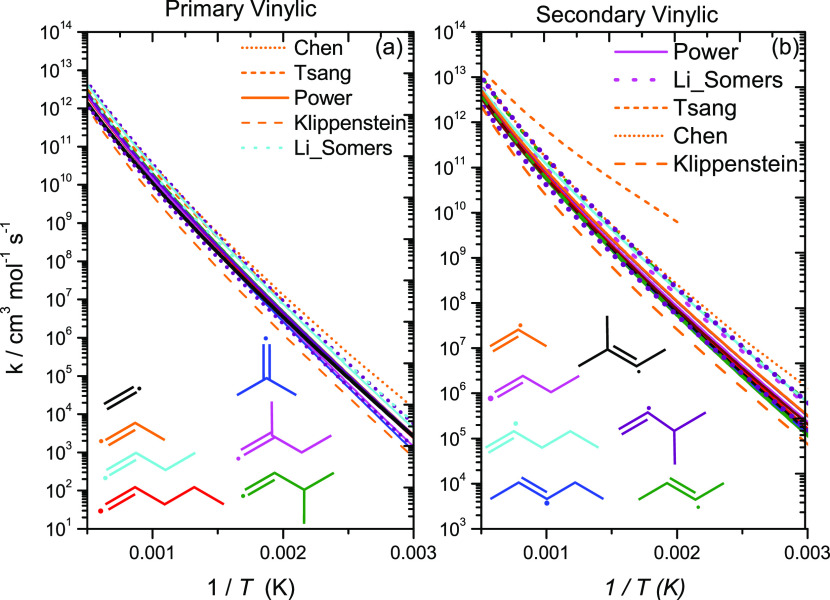
High-pressure limiting rate constants for H-atom abstraction from vinylic carbon sites on a per Ḣ atom basis.

For 2-alkenes, an average of our computed rate constants and the rate constant by Li et al.^2^ is taken as the recommended value. For comparison purposes, the rate constant calculated by Miller and Klippenstein^18^ was decreased by a factor of 2 since this was another reaction for which they altered the energy barrier. The altered rate constant agrees well with the rate constant calculated in the current work and with that from Chen et al.^20^ Moreover, for abstraction from the secondary allylic and primary vinylic carbon site, an average of our calculated rates and that by Li et al.^2^ is taken as the recommended rate constant. For abstraction from the secondary vinylic site, an average of our computed rate constants, Li et al., Chen et al., and Miller and Klippenstein is taken.^2,18,20^ A factor of 2 uncertainty is applied to these recommendations and is represented by dotted purple lines in Figures 12–14. For clarity reasons, the factor of 2 uncertainty is not shown in Figure 13a since there are two recommended rate constants.

### 3.3 Reactions of Alkyl Radicals

#### 3.3.1 Ethyl (Ċ_2_H_5_) Radical

Ethyl radicals are formed via Ḣ atom addition to ethylene (11.2 kJ mol^–1^). C–H β-scission of ethyl radicals can also occur with a barrier height of 157.7 kJ mol^–1^.

**Table 10:** Computed Energy Barriers, Heats of Reaction, and High-Pressure Limiting Rate Constant Fits for the Reactions of C_3_–C_4_ Alkyl Radicals^a^^,^^b^

reaction	Δ^‡^*H*_0K_	Δ*_r_H*_0K_	*A*	*n*	*E*_a_
nĊ_3_H_7_ ⇌ iĊ_3_H_7_	158.09	–13.86	2.22 × 10^05^	2.05	129.83
nĊ_3_H_7_ ↔ C_2_H_4_ + ĊH_3_	127.91	91.40	1.10 × 10^16^	–0.72	135.31
Ċ_4_H_9_-1 ⇌ Ċ_4_H_9_-2	162.78	–11.15	8.80 × 10^–05^	4.82	111.84
Ċ_4_H_9_-1 ↔ C_2_H_4_ + Ċ_2_H_5_	124.29	89.93	3.64 × 10^15^	–0.58	130.33
Ċ_4_H_9_-2 ↔ C_3_H_6_ + ĊH_3_	129.45	93.21	1.87 × 10^14^	–0.20	134.93
iĊ_4_H_9_ ⇌ Ċ_4_H_9_-t	150.21	–21.17	8.08 × 10^01^	3.03	116.48
iĊ_4_H_9_ ↔ C_3_H_6_ + ĊH_3_	129.86	86.81	1.46 × 10^17^	–0.91	137.99

a Units (*AT^n^* = s^–1^, energies = kJ mol^–1^).

b Fit between 298 and 2000 K.

#### 3.3.2 Propyl (nĊ_3_H_7_ and iĊ_3_H_7_) Radicals

Once nĊ_3_H_7_ radicals are formed via internal Ḣ atom addition to propene, they can undergo C–C β-scission to form ethylene and ĊH_3_ radicals with an energy barrier of 127.9 kJ mol^–1^ (Table 10), which is more favorable (by 30.2 kJ mol^–1^) than isomerization to iĊ_3_H_7_ radicals. They can also undergo C–H β-scission, with an energy barrier of 148.6 kJ mol^–1^. The iĊ_3_H_7_ radicals formed can undergo a Ḣ atom elimination reaction, with an energy barrier of 155.2 kJ mol^–1^.

#### 3.3.3 Butyl (Ċ_4_H_9_-1 and Ċ_4_H_9_-2) Radicals

**Figure 15:**
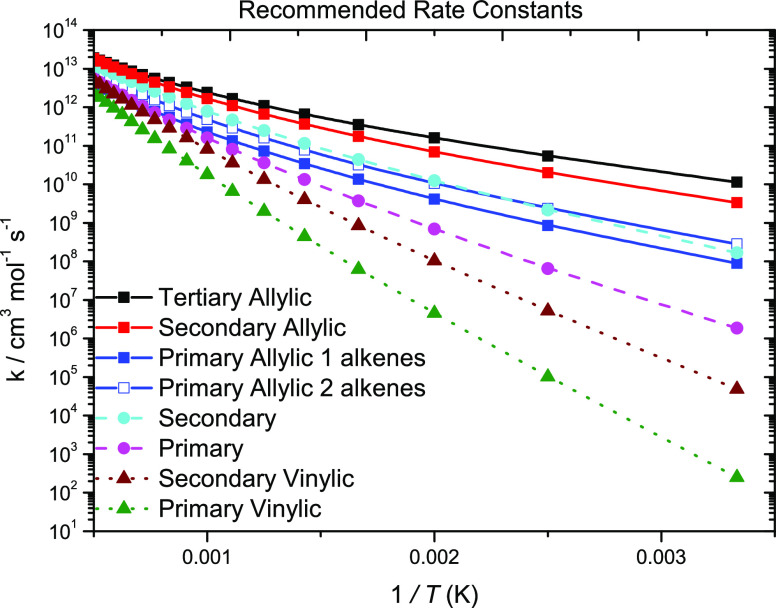
Rate constant recommendations for H-atom abstraction from C_2_–C_5_ alkenes. Solid lines, allylic; dashed lines, alkyl; and dotted lines, vinylic.

Ċ_4_H_9_-1 radicals are formed via internal Ḣ atom addition to 1-butene, while terminal addition leads to the formation of Ċ_4_H_9_-2 radicals. Ċ_4_H_9_-2 radicals are also formed through internal Ḣ atom addition to 2-butene. C–C β-scission of Ċ_4_H_9_-1 radicals can occur forming ethylene and Ċ_2_H_5_ radicals, with a barrier height of 124.3 kJ mol^–1^, which is more favorable (by 33.5 kJ mol^–1^) than isomerization to Ċ_4_H_9_-2 radicals. Additionally, C–H β-scission of Ċ_4_H_9_-1 radicals can occur with a barrier height of 152.5 kJ mol^–1^. Two transition states are available for the reaction Ċ_4_H_9_-1 ⇌ Ċ_4_H_9_-2, one occurring through a three-membered ring and the second one occurring through a four-membered ring, with barrier heights of 160.6 and 162.8 kJ mol^–1^, respectively. C–C β-scission of Ċ_4_H_9_-2 can also occur, forming propene and a ĊH_3_ radical, with an energy barrier of 129.45 kJ mol^–1^, while C–H β-scission of Ċ_4_H_9_-2 has a barrier height of 148.6 kJ mol^–1^ (Figure 15).

#### 3.3.4 Branched Butyl (iĊ_4_H_9_ and tĊ_4_H_9_) Radicals

Internal Ḣ atom addition to isobutene forms iĊ_4_H_9_ radicals, while terminal addition forms tĊ_4_H_9_ radicals. C–H β-scission of iĊ_4_H_9_ radicals can occur, with a barrier height of 145.6 kJ mol^–1^. However, C–C β-scission of iĊ_4_H_9_ radicals, forming propene and ĊH_3_ radicals is more favored, with a reaction barrier of 129.9 kJ mol^–1^. Isomerization of iĊ_4_H_9_ to tĊ_4_H_9_ occurs with a higher energy barrier of 150.2 kJ mol^–1^. C–H β-scission of tĊ_4_H_9_ can occur, with a barrier height of 151.7 kJ mol^–1^.

**Figure 16:**
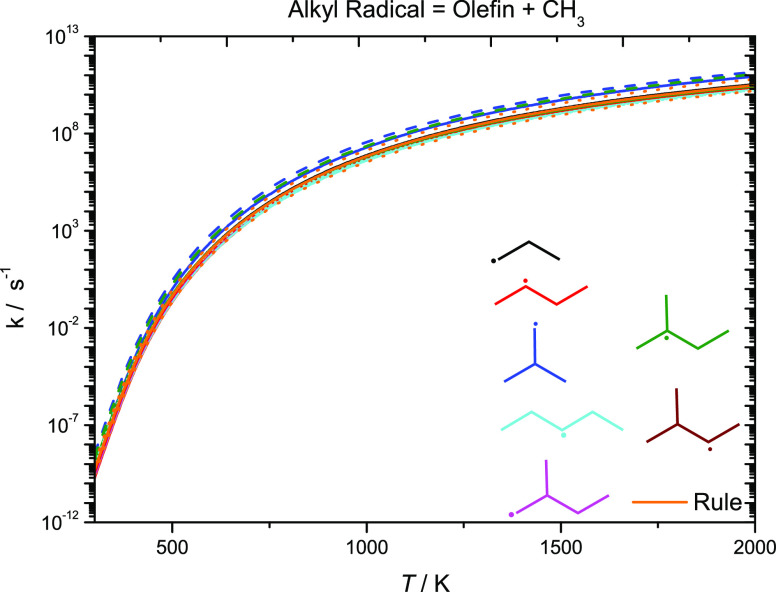
High-pressure limiting rate constants for alkyl radical decomposition, forming an olefin + ĊH_3_. Solid line, current work; dashed line, Curran; dotted line, Awan; and short-dotted line, Comandini.

Figure 16 presents high-pressure limiting rate constants for alkyl radical decomposition reactions forming an olefin + ĊH_3_. For comparison, rate constants for alkyl radical decomposition from our previous work on C_5_ alkenes in addition to other literature sources^4,59,61−63^ are plotted. The rate constant for the reaction iĊ_4_H_9_ ↔ C_3_H_6_ + ĊH_3_ recommended by Curran^59^ is a factor of 2.74–1.67 times faster than that calculated in this work in the temperature range 500–2000 K. With the exception of this reaction, all other rate constants calculated in this work for alkyl radicals, leading to the formation of an olefin and a ĊH_3_ radical are within a factor of 1.55 of our computed rate constant for nĊ_3_H_7_ ↔ C_2_H_4_ + ĊH_3_ over the temperature range 298–2000 K. The rate constant calculated in this work for iĊ_4_H_9_ ↔ C_3_H_6_ + ĊH_3_ is a factor of 2.54–3.08 times faster than nĊ_3_H_7_ ↔ C_2_H_4_ + ĊH_3_. This may be due to the fact that iĊ_4_H_9_ radicals have three degenerate sites for C–C β-scission to take place.

**Figure 17:**
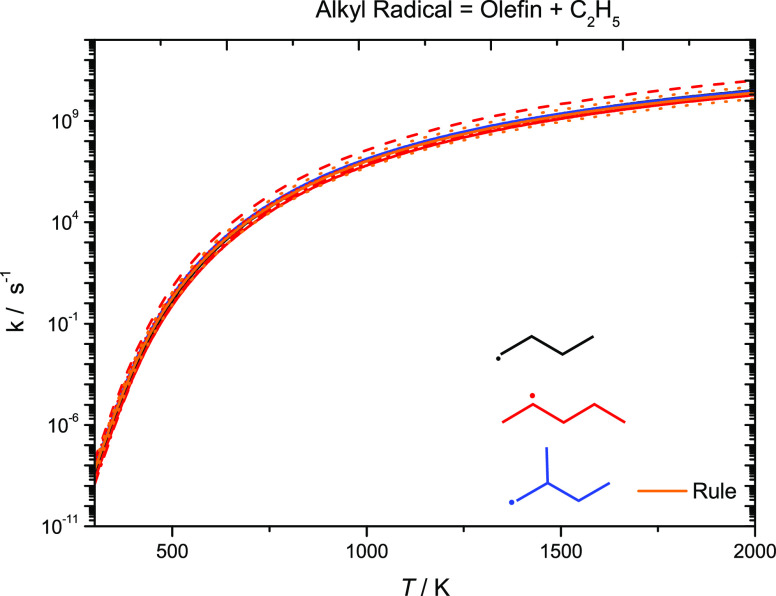
High-pressure limiting rate constants for alkyl radical decomposition, forming an olefin + Ċ_2_H_5_. Solid line, current work; dashed line, Curran; dotted line, Awan; short-dotted line, Comandini; and dashed-dotted line, Jitariu.

Reasonable agreement is observed for the reactions of alkyl radicals forming an olefin and Ċ_2_H_5_ radicals calculated previously and in this work. In Figure 17, the rate constant recommendation by Curran^59^ for the reaction Ċ_5_H_11_-2 ↔ C_3_H_6_ + Ċ_2_H_5_ is the fastest in comparison to the other analogous reactions. The Curran recommendation^59^ is a factor of ca. 9.5–5.0 times faster than our calculated rate constant for Ċ_5_H_11_-2 ↔ C_3_H_6_ + Ċ_2_H_5_ calculated previously over the temperature range 300–2000 K.^4^ The value from Awan and Comandini^61,62^ is a factor of ∼3.4 times faster than our previous work^4^ for the same reaction at 500 K, with the rate constants converging at higher temperatures. Jitariu et al.^63^ are in excellent agreement with our previous work for the reaction Ċ_5_H_11_-2 ↔ C_3_H_6_ + Ċ_2_H_5_,^4^ with the rate constants being within a factor of ∼1.3. The rate constant for the reaction aĊ_5_H_11_ ↔ C_3_H_6_ + Ċ_2_H_5_ calculated in our most recent study^5^ is a factor of ∼3 times faster at 500 K than our calculated rate constant for Ċ_5_H_11_-2 ↔ C_3_H_6_ + Ċ_2_H_5_.^4^ An energy barrier difference of 1.9 kJ mol^–1^ accounts for a factor of 1.6 of this difference. For the decomposition of an alkyl radical forming an olefin and an ethyl radical, an average of the rate constants calculated in our current and previous studies^4,5^ as well as those by Awan, Comandini, and Jitariu^,61−63^ is taken, with a factor of 2 of the recommended represented as orange dotted lines.

**Figure 18:**
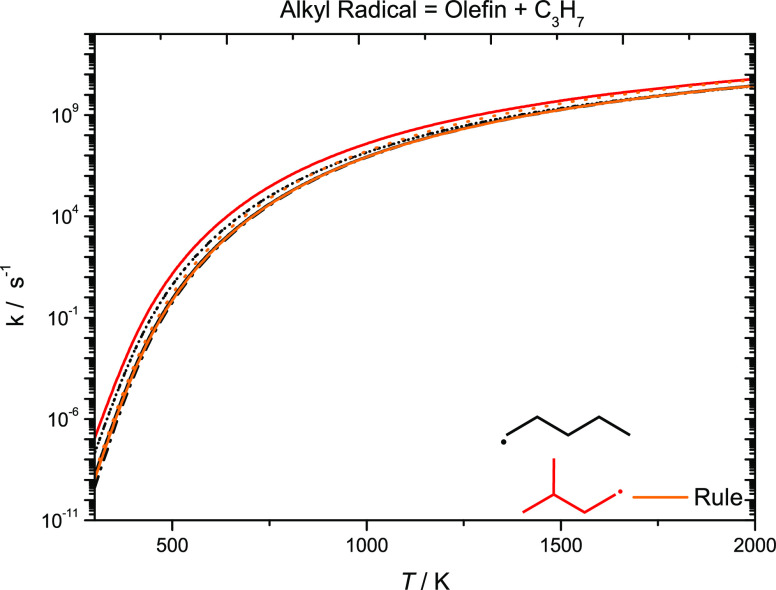
High-pressure limiting rate constants for alkyl radical decomposition, forming an olefin + Ċ_3_H_7_. Solid line, current work; dotted line, Awan; short-dotted line, Comandini; and dashed-dotted line, Jitariu.

Figure 18 presents rate constant comparisons for alkyl radical decomposition forming olefin and propyl radicals. The reactions Ċ_5_H_11_-1 ↔ C_2_H_4_ + nĊ_3_H_7_ and dĊ_5_H_11_ ↔ C_2_H_4_ + iĊ_3_H_7_ are plotted for comparison. The rate constants for the reaction Ċ_5_H_11_-1 ↔ C_2_H_4_ + nĊ_3_H_7_ by Awan,^61^ Comandini,^62^ and Jitariu et al.^63^ are in good agreement with our previously calculated rate constant.^4^ At 500 K, the values from Awan^61^ and Comandini^62^ are a factor of ∼4.5 times faster than our calculated rate constant for this reaction at 500 K, with the rate constants converging at high temperatures. The difference of 7.76 kJ mol^–1^ in the energy barrier accounts for the observed difference. Larger differences are observed between the values calculated in this work and by Awan and Comandini at temperatures below 500 K; therefore, the recommended rate constant for Ċ_5_H_11_-1 ↔ C_2_H_4_ + *n*Ċ_3_H_7_ is taken as an average of the rate calculated in the current work and by Jitariu et al.^63^ The rate constant by Jitariu et al. is in excellent agreement with our calculated rate constant for the same reaction.^4^ Our calculated rate constant for dĊ_5_H_11_ ↔ C_2_H_4_ + iĊ_3_H_7_ is also plotted in this graph, which is taken as the recommended rate constant for alkyl radical decomposition forming an olefin and an iĊ_3_H_7_ radical (Table 11).

**Table 11:** Recommended Rate Constants for Alkyl Radical Decomposition Forming an Olefin + Radical^a^

class	*A*	*n*	*E*_a_	uncertainty bounds (upper, lower)
alkyl radical ↔ olefin + ĊH_3_	2.54 × 10^10^	1.04	30 573	2.86, 3.71
alkyl radical ↔ olefin + Ċ_2_H_5_	5.20 × 10^11^	0.57	29 308	1.34, 3.11
alkyl radical ↔ olefin + nĊ_3_H_7_	9.62 × 10^11^	0.55	30 678	1.61, 2.60
alkyl radical ↔ olefin + iĊ_3_H_7_	6.87 × 10^12^	0.31	28 225	

a (*AT^n^* = s^–1^, energies = cal mol^–1^). Fit between 300 and 2000 K.

## 4.0 Detailed Kinetic Modeling

**Figure 19:**
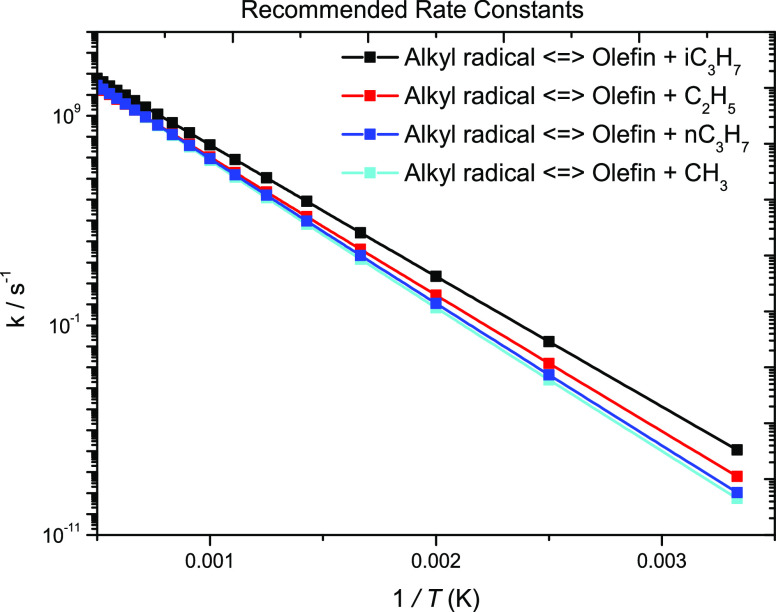
Rate constant recommendations for alkyl radical decomposition to olefin + radical.

All simulations were performed using Chemkin-Pro assuming a constant-volume homogeneous batch reactor. As described in our previous study of the pentene isomers,^5^ test computations implied that the high-pressure limiting rate constant for external Ḣ atom addition to 2M1B was overestimated by a factor of 2–3, which is also in line with the variational effect observed by Jasper and Hansen^64^ for Ḣ atom addition to high-molecular-weight species. To assess the influence of variational effects on predictions of experimental data, indicative simulations are carried out by systematically reducing the rate constants for Ḣ atom addition by a factor of 2 in the RRKM/ME model and recomputing *k*(*T*,*p*). The approximate variational calculation results from the current work for the alkene + Ḣ systems have been included in NUIGMech1.1, which includes our results from our previous studies of the pentene isomers.^4,5^ The updated model, NUIGMech1.2, is used to simulate the recent results from a pyrolysis study of 1-alkenes using the NUIG single-pulse shock tube^32^ and is represented by solid lines. Dashed lines represent model predictions of NUIGMech1.1. The update to the pyrolysis reactions between the two models is solely from the present work. Improvements in species mole fractions are observed, particularly for 2-butene and isobutene pyrolysis.^35^ The supporting information contains PLOG fits for both the approximate variational results and the original unadjusted results (Figure 19).

### 4.1 Ethylene Pyrolysis

**Figure 20:**
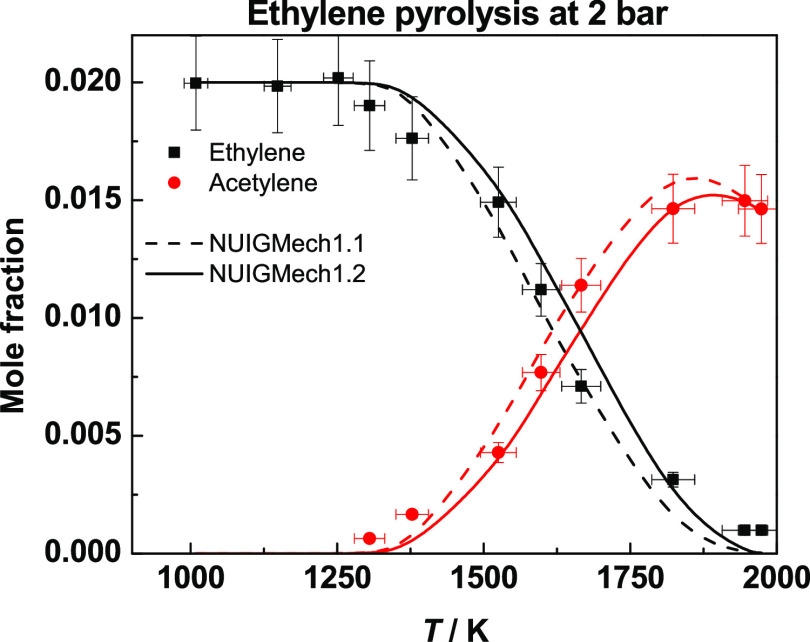
Species profiles for ethylene pyrolysis at 2 bar. Dashed lines represent NUIGMech1.1, and solid lines represent NUIGMech1.2.

Figure 20 presents species profiles for ethylene pyrolysis at 2 bar.^32^ The reaction path analysis was already described by Nagaraja et al.^32^ so we shall be brief here. H-atom abstraction by Ḣ atoms from ethylene leads to the production of vinyl radicals, with vinyl radicals decomposing to acetylene + Ḣ. Through the incorporation of the rate constants calculated in this work (NUIGMech1.2), there is a slight improvement in the species profiles for both ethylene and acetylene. The rate constant for H-atom abstraction from ethylene is approximately a factor of 2 slower, which reduces the amount of vinyl radical produced, which in turn decreases the production of acetylene and Ḣ atoms.

### 4.2 Propene Pyrolysis

**Figure 21:**
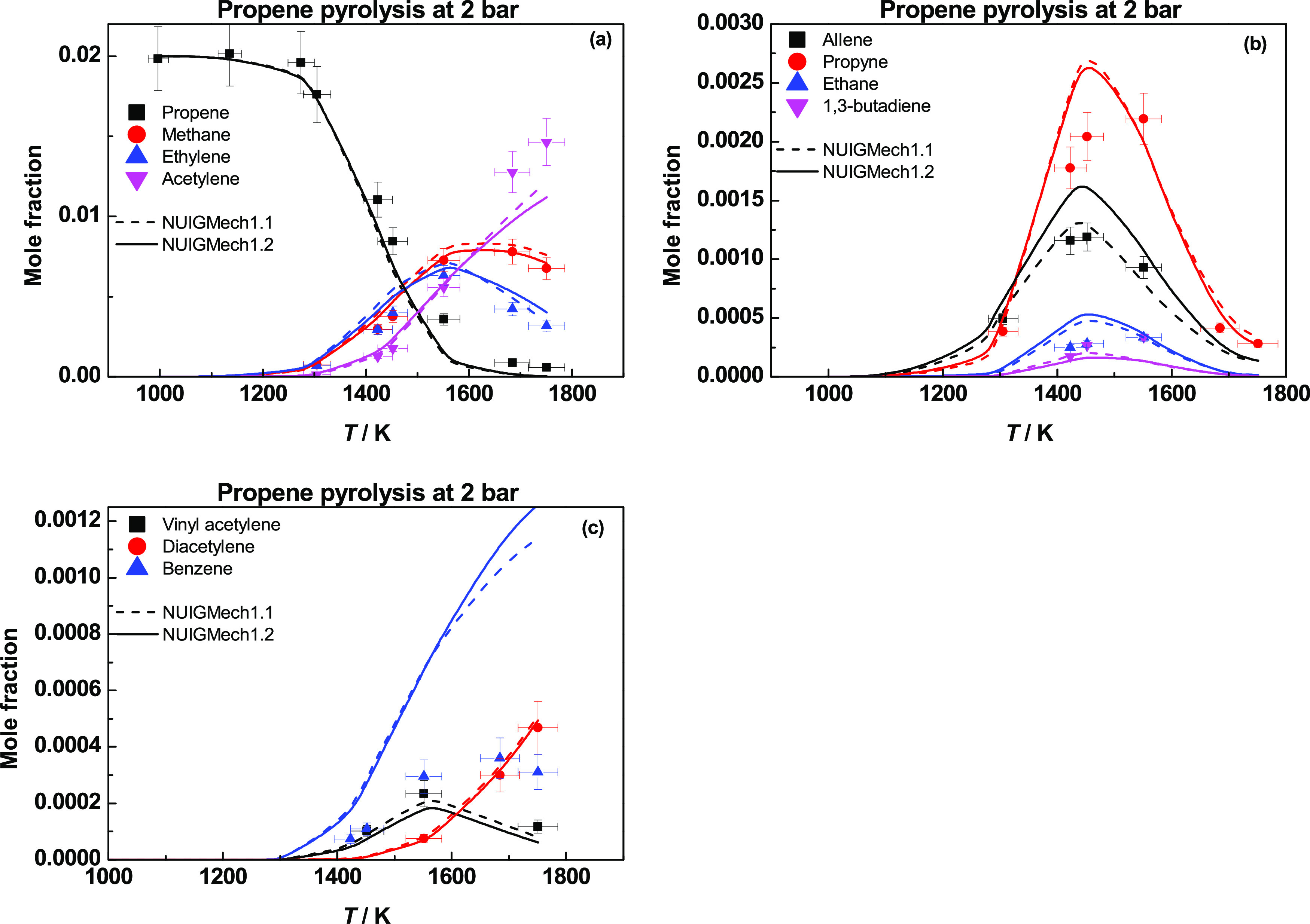
Species profiles for propene pyrolysis at 2 bar. Dashed lines represent NUIGMech1.1, and solid lines represent NUIGMech1.2.

Figure 21 presents species profiles for propene pyrolysis at 2 bar.^32^ Both Ḣ atom addition and abstraction reactions are the main consumption pathways for propene. Ḣ atom addition to propene and the subsequent decomposition of propyl radical lead to the formation of ethylene and a methyl radical. The abstraction of an allylic H-atom by Ḣ atoms or ĊH_3_ radicals leads to the formation of allyl and H_2_ and CH_4_. Allyl radicals are converted to allene, which subsequently isomerizes to propyne or undergoes H-atom abstraction to form propargyl radicals, which in turn produces benzene. Acetylene is formed by the decomposition of vinyl radicals, the reaction of Ḣ atoms with allene and propyne, and the β-scission of propen-1-yl radical.

### 4.3 1-Butene Pyrolysis

**Figure 22:**
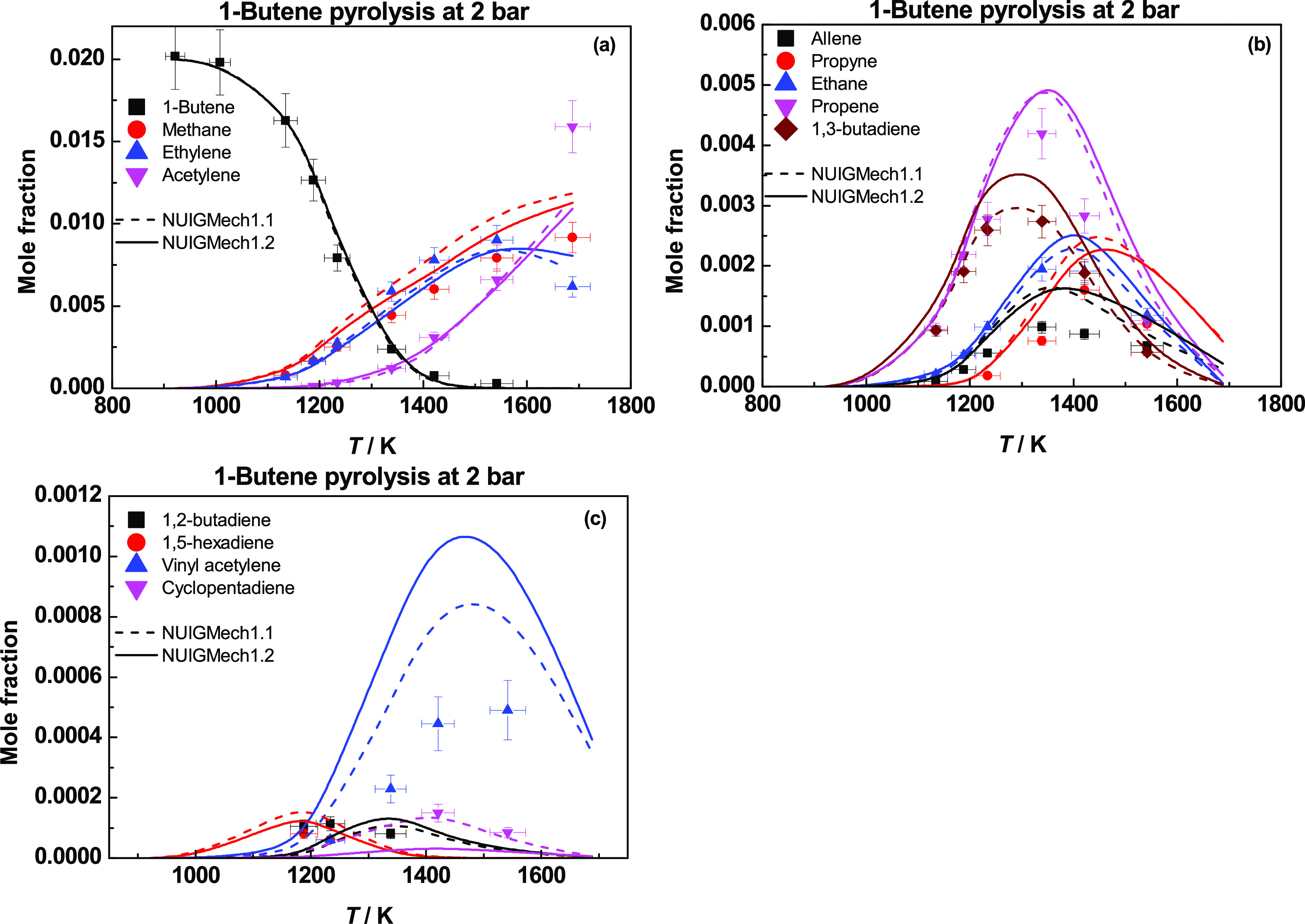
Species profiles for 1-butene pyrolysis at 2 bar. Dashed lines represent NUIGMech1.1, and solid lines represent NUIGMech1.2

Figure 22 presents species profiles for 1-butene pyrolysis at 2 bar.^32^ The pyrolysis chemistry is quite similar to that of propene, with both Ḣ atom addition and abstraction reactions being important pathways. H-atom addition to 1-butene produces propene and a ĊH_3_ radical and ethylene and a Ċ_2_H_5_ radical via two chemically activated pathways. Ethyl radicals decompose to ethylene + Ḣ. Hydrogen atom abstraction by Ḣ or ĊH_3_ leads to the formation of Ċ_4_H_7_1–3, which in turn forms 1,3-butadiene. Methane is formed primarily by H-atom abstraction by ĊH_3_ radicals from the fuel and other stable species. Acetylene is mainly produced by the decomposition of vinyl radicals and the reactions of Ḣ atoms with allene and propyne.

### 4.4 Trans 2-Butene Pyrolysis

**Figure 23:**
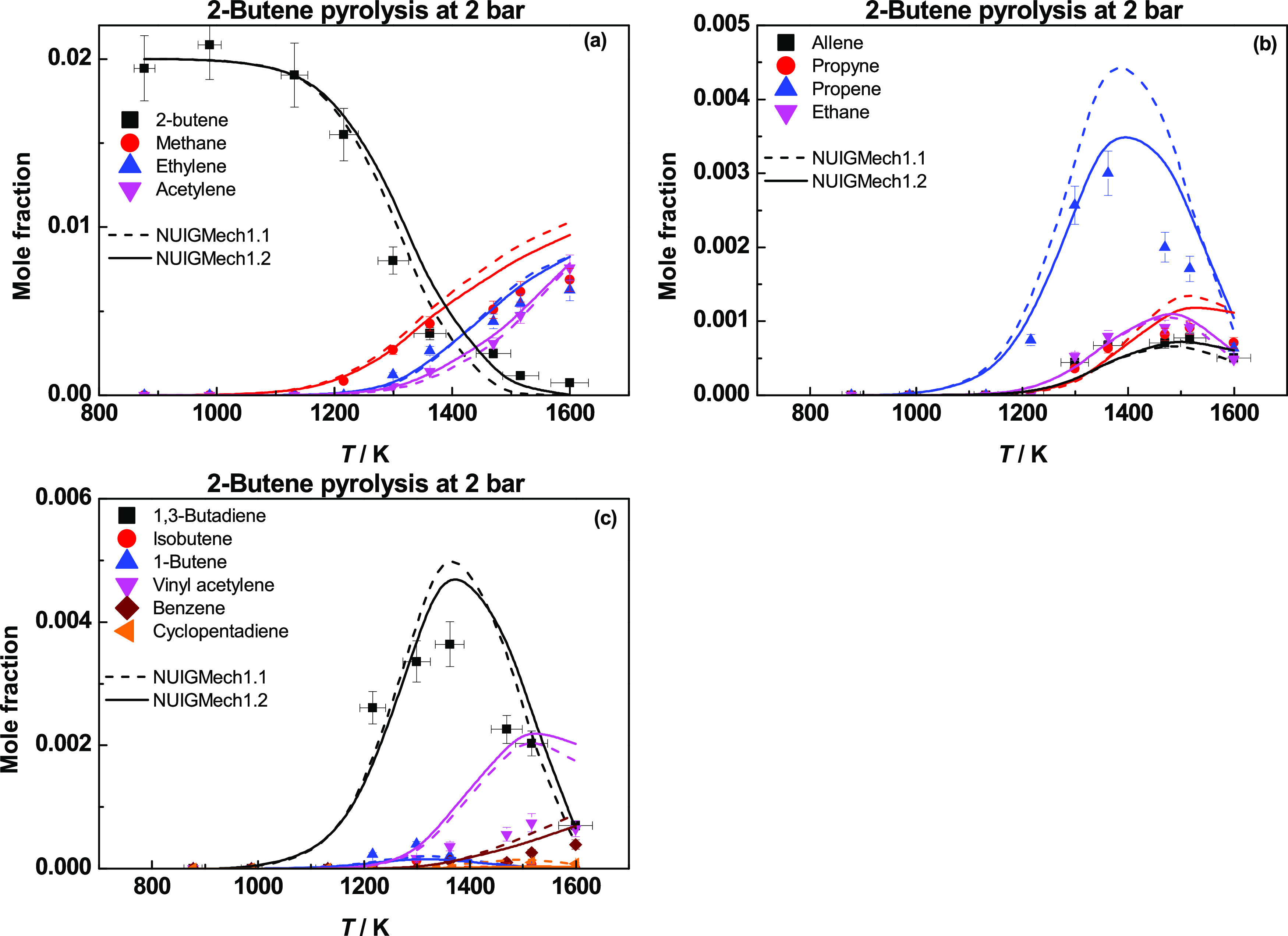
Species profiles for 2-butene pyrolysis at 2 bar. Dashed lines represent NUIGMech1.1, and solid lines represent NUIGMech1.2.

Figure 23 presents species profiles for 2-butene pyrolysis at 2 bar.^35^ Again, the pyrolysis chemistry is quite similar to that of propene and 1-butene. Ḣ atom addition to 2-butene forms propene and ĊH_3_ radicals through a chemically activated pathway. H-atom abstraction by Ḣ or ĊH_3_ leads to the formation of Ċ_4_H_7_1-3, which in turn forms 1,3-butadiene. The rate constant for H-atom abstraction from 2-butene forming Ċ_4_H_7_1-3 calculated in this work is a factor of 2.5 times slower than that used in NUIGMech1.1, which in turn reduces the species mole fraction of 1,3-butadiene. For the propene species profiles, there is an improvement in the predictions through the incorporation of the calculations computed in the current work. The production of propene, as previously stated, comes from the chemically activated pathway of Ḣ atom addition to 2-butene. The rate constants in NUIGMech1.1 are based on QRRK/MSC estimates and are approximately a factor of ∼7 times faster than those in NUIGMech1.2 at 1400 K. Again, methane is mainly produced by H-atom abstraction by ĊH_3_ from the fuel and other stable species. Acetylene is mainly produced by the decomposition of vinyl radicals and the reactions of Ḣ atoms with allene and propyne.

### 4.5 Isobutene Pyrolysis

**Figure 24:**
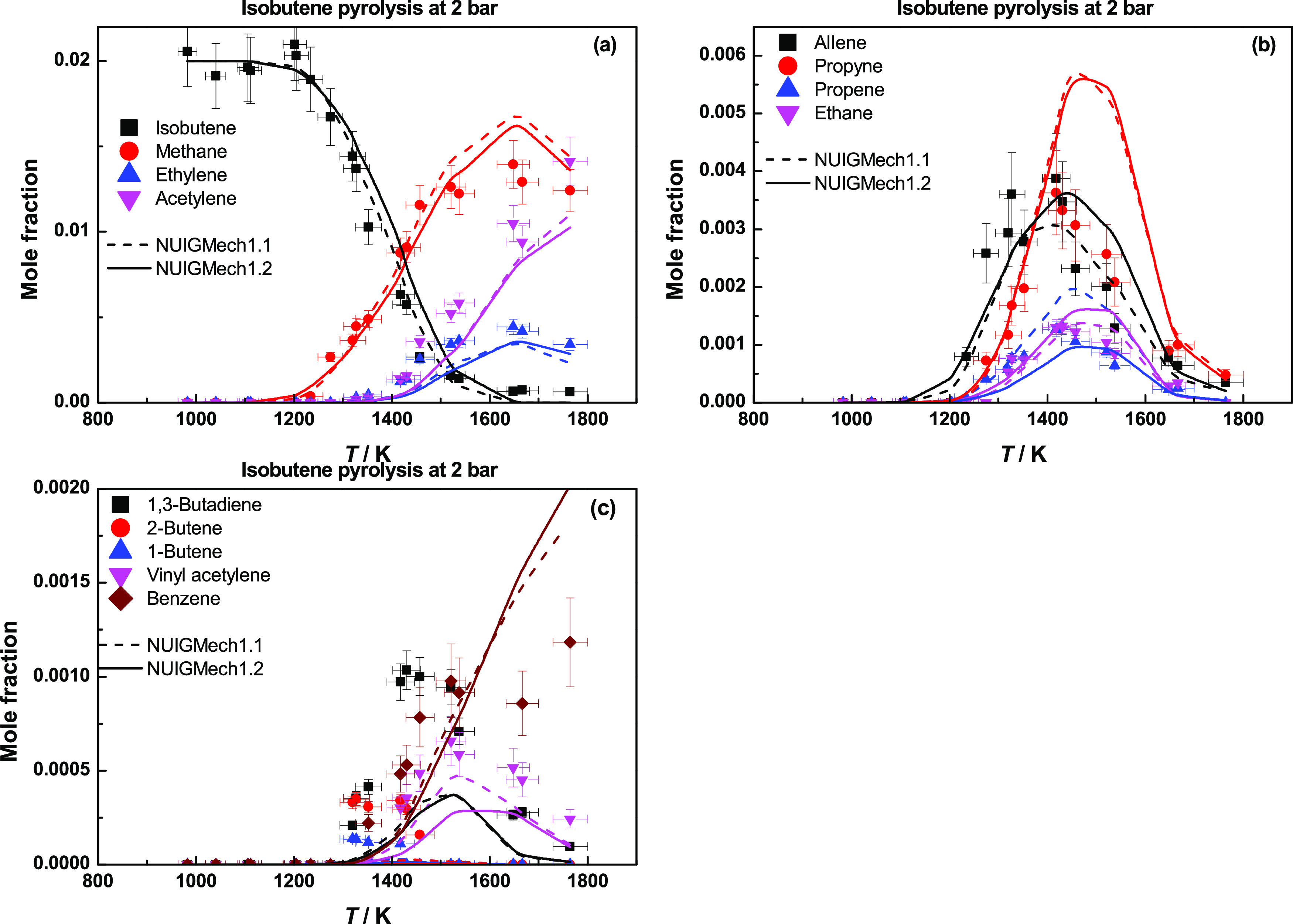
Species profiles for isobutene pyrolysis at 2 bar. Dashed lines represent NUIGMech1.1, and solid lines represent NUIGMech1.2.

Figure 24 presents species profiles for isobutene pyrolysis at 2 bar.^35^ Ḣ atom abstraction from isobutene leads to the formation of iĊ_4_H_7_ radicals, which decompose to produce allene and ĊH_3_ radicals. The resulting allene then isomerizes to propyne. Propene is primarily formed through the chemically activated pathway of Ḣ atom reaction to isobutene. There is an improvement in the propene predictions with the current model, due to the rate constant for the chemically activated pathway of Ḣ atom addition to isobutene being approximately a factor of 7 times slower at 1400 K.

## 5.0 Chemically Activated Pathways

### 5.1 Effect of Pressure

**Figure 25:**
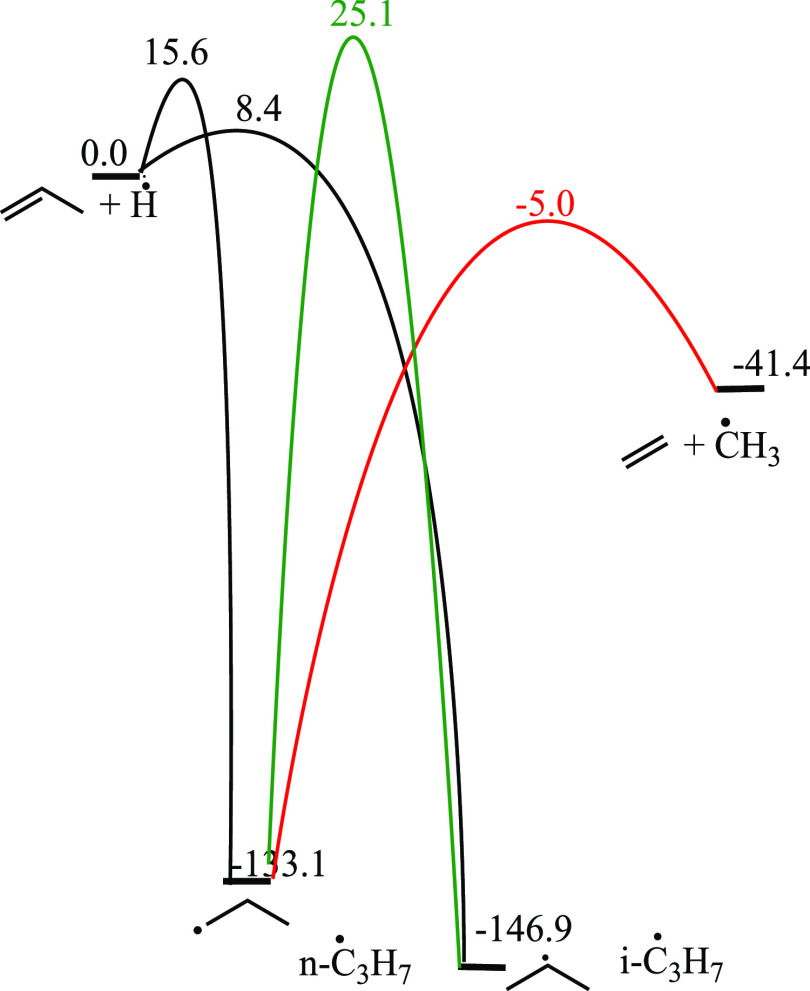
Potential energy surface for Ḣ-atom addition reactions of propene. Energies in kJ mol^–1^.

From the simulations, it is observed that the chemically activated pathways for the reaction of Ḣ atoms with alkenes are important in capturing the species profiles of the products during pyrolysis and oxidation (Figure 25). Taking propene as an example, which is described in Figure 4, the formation of stabilized iĊ_3_H_7_ radicals through the reaction of Ḣ atoms with propene dominates at temperatures up to 800, 1000, 1200, and 1500 and pressures of 0.1, 1, 10, and 100 atm, respectively. The chemically activated pathway C_3_H_6_ + Ḣ ↔ [nĊ_3_H_7_]* ↔ C_2_H_4_ + ĊH_3_ then dominates the reaction flux at higher temperatures. At 1000 atm, the formation of stabilized iĊ_3_H_7_ radicals dominates over the entire temperature range.

At 1000 K and 0.1 atm, 70% of the reaction flux goes through this chemically activated pathway for C_3_H_6_ + Ḣ. However, as the pressure increases, this percentage reduces, and the stabilization reaction channel becomes more favorable. The percentages of reaction flux going through this chemically activated pathway are 41, 20, 7, and 2% for pressures of 1, 10, 100, and 1000 atm, respectively. It is therefore important to have accurate rate constants for the chemically activated pathways on these potential energy surfaces to predict the species mole fractions across a wide range of temperatures and pressures. Below are a list of some of the chemically activated pathways forming some of the major products of pyrolysis calculated in the current study and in previous ones.^4,5^

- C_3_H_6_ + Ḣ ↔ [nĊ_3_H_7_]* ↔ C_2_H_4_ + ĊH_3_
- C_4_H_8_-1 + Ḣ ↔ [Ċ_4_H_9_-1]* ↔ C_2_H_4_ + Ċ_2_H_5_
- C_4_H_8_-1 + Ḣ ↔ [Ċ_4_H_9_-2]* ↔ C_3_H_6_ + ĊH_3_
- C_4_H_8_-2 + Ḣ ↔ [Ċ_4_H_9_-2]* ↔ C_3_H_6_ + ĊH_3_
- iC_4_H_8_ + Ḣ ↔ [iĊ_4_H_9_]*↔ C_3_H_6_ + ĊH_3_
  Moreover, chemically activated pathways were also found to be important for the reactions of Ḣ with the pentene isomers in our previous studies.^4,5^
- C_5_H_10_-1 + Ḣ ↔ [Ċ_5_H_11_-1]* ↔ C_2_H_4_ + n-Ċ_3_H_7_
- C_5_H_10_-1 + Ḣ ↔ [Ċ_5_H_11_-2]* ↔ C_3_H_6_ + Ċ_2_H_5_
- C_5_H_10_-2 + Ḣ ↔ [Ċ_5_H_11_-2]* ↔ C_3_H_6_ + Ċ_2_H_5_
- C_5_H_10_-2 + Ḣ ↔ [Ċ_5_H_11_-3]* ↔ C_4_H_8_-1 + ĊH_3_
- aC_5_H_10_ + Ḣ ↔ [aĊ_5_H_11_]* ↔ C_3_H_6_ + Ċ_2_H_5_
- aC_5_H_10_ + Ḣ ↔ [aĊ_5_H_11_]* ↔ C_4_H_8_-1 + ĊH_3_
- aC_5_H_10_ + Ḣ ↔ [bĊ_5_H_11_]* ↔ iC_4_H_8_ + ĊH_3_
- bC_5_H_10_ + Ḣ ↔ [bĊ_5_H_11_]* ↔ iC_4_H_8_ + ĊH_3_
- bC_5_H_10_ + Ḣ ↔ [cĊ_5_H_11_]* ↔ C_4_H_8_-2 + ĊH_3_
- cC_5_H_10_ + Ḣ ↔ [cĊ_5_H_11_]* ↔ C_4_H_8_-2 + ĊH_3_
- cC_5_H_10_ + Ḣ ↔ [dĊ_5_H_11_]* ↔ C_2_H_4_ + iĊ_3_H_7_

### 5.2 Effect of Molecular Size

**Figure 26:**
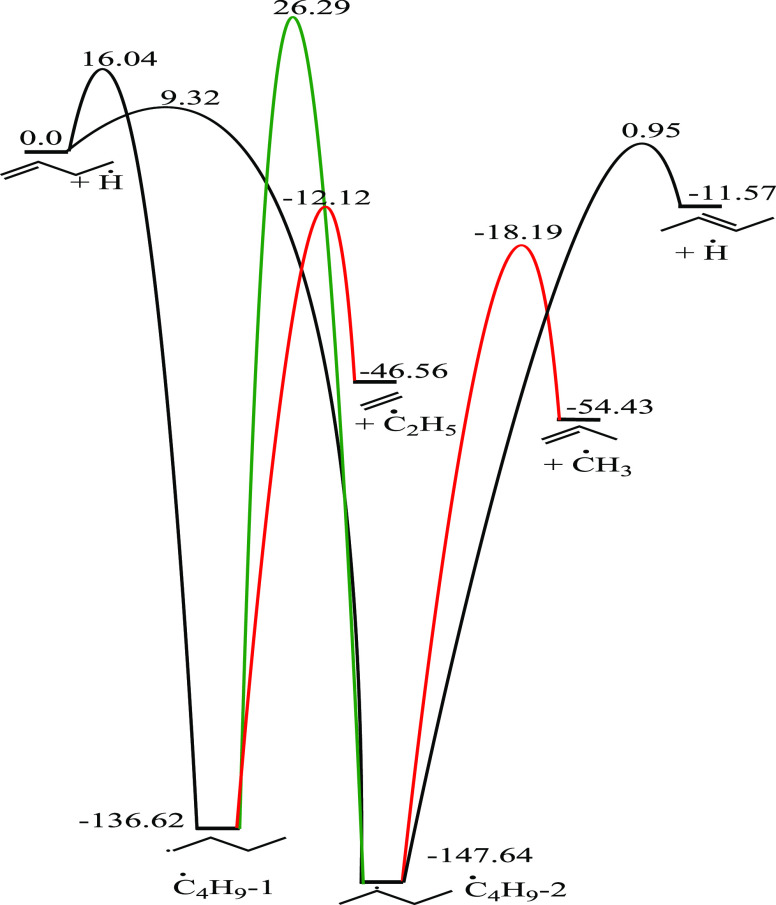
Potential energy surface for Ḣ-atom addition reactions of 1- and 2-butene. Energies in kJ mol^–1^.

As the size of the molecule increases from propene to 1-butene, the effect of chemical activation becomes greater, especially at lower pressures. At 1000 K and 0.1 atm, for 1-butene, 99% of the reaction flux proceeds through the chemically activated pathways compared to 70% for propene (Figure 26).

**Figure 27:**
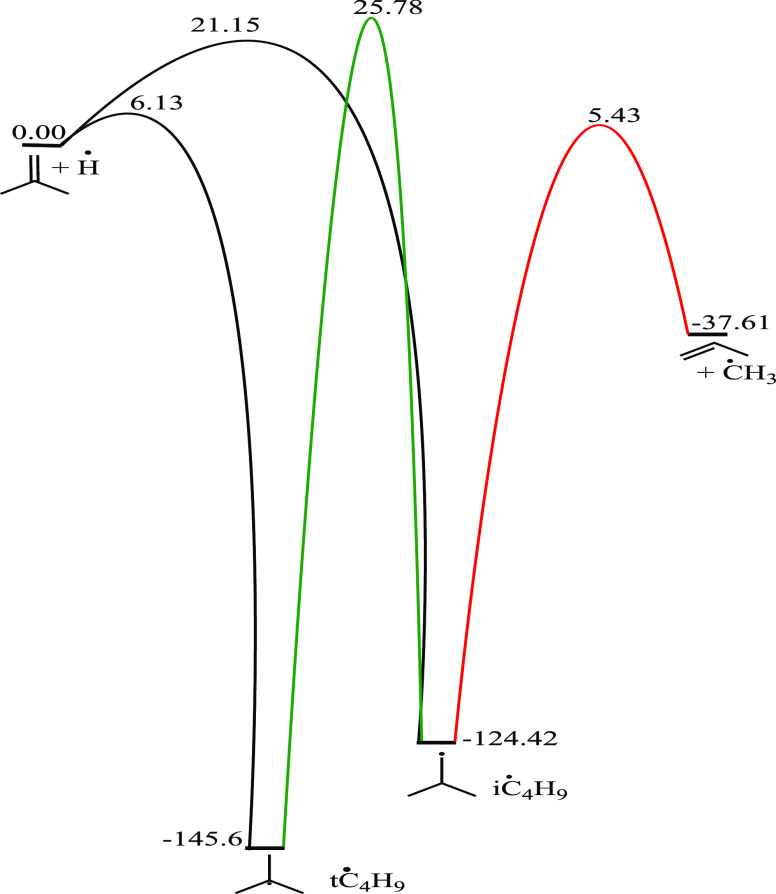
Potential energy surface for Ḣ-atom addition reactions of isobutene. Energies in kJ mol^–1^.

For the pentene isomers, it was shown that >95% of the reaction flux proceeds through the chemically activated pathways at 1000 K and 0.1 atm,^4,5^ which is similar to butene. As the pressure increases, this percentage reduces to 93, 65, 25, and 4% at pressures of 1, 10, 100, and 1000 atm, respectively, for 1-butene, compared to 41, 20, 7, and 2% for propene. The formation of stabilized Ċ_4_H_9_-2 radicals through the reaction of Ḣ atoms with 1-butene then dominates, which can be seen in Figure 6a. A similar situation prevails for 2-butene (Figure 6b), where 98, 87, 57, 20, and 3% proceed through chemical activation at 0.1, 1, 10, 100, and 1000 atm, respectively. In the case of isobutene, 33% of the reaction flux goes through the chemically activated pathways at 1000 K and 0.1 atm, with the formation of stabilized tĊ_4_H_9_ radicals then dominating the reaction flux. It is not until a temperature of 1200 K is reached that chemical activation is considerable, accounting for 74% at 0.1 atm (Figure 6c). It is observed that the effect of chemical activation becomes greater as the molecular size increases from propene to butene. However, as the molecular size increases from butene to pentene, the effect of chemical activation is similar (Figure 27).

## 6 Conclusions

To contribute to the development of combustion models, a hierarchical set of rate constants for the reactions of Ḣ atom with C_2_–C_5_ alkenes, and the subsequent C–C and C–H β-scission and Ḣ atom transfer reactions using the same level of theory now exist. The reactions for the linear and branched C_5_ alkenes were performed in our previous studies, while calculations for C_2_–C_4_ species are performed in the current work. Thermochemical data are calculated as a function of temperature, with enthalpies of formation determined from an isodesmic network, which is built upon benchmark literature data and electronic structure calculations. High-pressure limiting and temperature- and pressure-dependent rate constants are calculated using RRKM theory with a 1D master equation (ME) analysis. Rate constant recommendations for Ḣ atom addition/abstraction and alkyl radical decomposition are proposed and serve as a useful tool in mechanisms for larger alkenes for which calculations do not exist.

As mentioned in our earlier work,^5^ test computations implied that the high-pressure limiting rate constant for Ḣ atom addition were overestimated by a factor of 2–3, which is also in line with the variational effect observed by others for Ḣ atom addition reactions to high-molecular-weight species.^64^ To determine the influence of variational effects on model predictions, indicative simulations are carried out by systematically reducing the rate constants for Ḣ atom addition by a factor of 2 in the RRKM/ME model and recomputing *k*(*T*,*p*). Similarly to our earlier work,^5^ it is found that the chemically activated pathways for Ḣ atom addition to alkenes, as well as their abstraction reactions, are found to be important in capturing the species profiles of the products from pyrolysis. Although a good agreement is observed between our model predictions and experiment, future work should consider to address VTST, the treatment of multidimensional torsions, and anharmonic effects with the aim of developing a more comprehensive RRKM/ME model for combustion modeling.

## Supporting Information Available

MESS input and output files and thermochemical values in NASA polynomial format and Chemkin format PLOG rate constant fits (ZIP)
Symmetry factors for reactants and transition states prior to symmetry-uncorrected rate constants (Table S1) (PDF)
