# Supplementary material for: Hierarchical Study of the Reactions of Hydrogen Atoms with Alkenes: A Theoretical Study of the Reactions of Hydrogen Atoms with C2–C4 Alkenes
Source: J Phys Chem A. 2021 Jun 8;125(23):5124–45. doi: 10.1021/acs.jpca.1c03168 (PMC8279655; doi:10.1021/acs.jpca.1c03168)
Supplement: Supplementary file 1 — jp1c03168_si_001.zip [file jp1c03168_si_001.zip › C2-C4_ALKENES+H_SUPPORTING_INFO/Symmetry_correction.docx]

**Supporting Information : A Hierarchical Study of the Reactions of Hydrogen atoms with Alkenes: A Theoretical Study of the Reaction of Hydrogen atoms with C_2_ – C_4_ Alkenes**

Jennifer Power^1^, Kieran P. Somers^1^, Shashank S. Nagaraja^1^, Henry J. Curran^1^

^1^Combustion Chemistry Centre, School of Chemistry, Ryan Institute, MaREI, National University of Ireland, Galway, Galway H91TK33, Ireland

Corresponding author: [henry.curran@nuigalway.ie](mailto:henry.curran@nuigalway.ie)

**Table S1. Symmetry factors for reactants and transition states prior to symmetry uncorrected rate constants**

| Reactant | Product | σ Reactant | σ Transition state | Symmetry corrected / symmetry uncorrected rate constants |
| --- | --- | --- | --- | --- |
| C_2_H_4_ | C_2_H_5_ | 4 | 2 | 2 |
| C_3_H_6_ | nC_3_H_7_ | 1 | 0.5 | 2 |
|  | iC_3_H_7_ | 1 | 0.5 | 2 |
| C_4_H_8_-1 | C_4_H_9_-1 | 1 | 0.5 | 2 |
|  | C_4_H_9_-2 | 1 | 0.5 | 2 |
| C_4_H_8_-2 | C_4_H_9_-2 | 2 | 0.5 | 4 |
| C_5_H_10_-1 | C_5_H_11_-1 | 1 | 0.5 | 2 |
|  | C_5_H_11_-2 | 1 | 0.5 | 2 |
| C_5_H_10_-2 | C_5_H_11_-2 | 1 | 0.5 | 2 |
|  | C_5_H_11_-3 | 1 | 0.5 | 2 |
| IC_4_H_8_ | iC_4_H_9_ | 2 | 1 | 2 |
|  | tC_4_H_9_ | 2 | 1 | 2 |
| 2M1B | aC_5_H_11_ | 1 | 0.5 | 2 |
|  | bC_5_H_11_ | 1 | 0.5 | 2 |
| 2M2B | bC_5_H_11_ | 1 | 0.5 | 2 |
|  | cC_5_H_11_ | 1 | 0.5 | 2 |
| 3M1B | cC_5_H_11_ | 1 | 0.5 | 2 |
|  | dC_5_H_11_ | 1 | 0.5 | 2 |
