# Supplementary material for: Hierarchical Study of the Reactions of Hydrogen Atoms with Alkenes: A Theoretical Study of the Reactions of Hydrogen Atoms with C2–C4 Alkenes
Source: J Phys Chem A. 2021 Jun 8;125(23):5124–45. doi: 10.1021/acs.jpca.1c03168 (PMC8279655; doi:10.1021/acs.jpca.1c03168)
Supplement: Supplementary file 2 — jp1c03168_si_002.pdf [file jp1c03168_si_002.pdf]

Supporting Information : A Hierarchical Study of the Reactions of Hydrogen atoms to Alkenes: A Theoretical Study of the Reaction of Hydrogen atoms with $C_2 – C_4$ Alkenes

Jennifer Power[1], Kieran Somers[1], Shashank Nagaraja[1], Henry J. Curran[1]

[1]Combustion Chemistry Centre, School of Chemistry, Ryan Institute, MaREI, National University of Ireland, Galway, Galway H91TK33, Ireland

Corresponding author: henry.curran@nuigalway.ie

Table S1. Symmetry factors for reactants and transition states prior to symmetry uncorrected rate constants

Reactant	Product	σ Reactant	σ Transition state	Symmetry corrected / symmetry uncorrected rate constants
C_2H_4	C_2H_5	4	2	2
C_3H_6	nC_3H_7	1	0.5	2
	iC_3H_7	1	0.5	2
C_4H_8-1	C_4H_9-1	1	0.5	2
	C_4H_9-2	1	0.5	2
C_4H_8-2	C_4H_9-2	2	0.5	4
C_5H_{10}-1	C_5H_{11}-1	1	0.5	2
	C_5H_{11}-2	1	0.5	2
C_5H_{10}-2	C_5H_{11}-2	1	0.5	2
	C_5H_{11}-3	1	0.5	2
IC_4H_8	iC_4H_9	2	1	2
	tC_4H_9	2	1	2
2M1B	aC_5H_{11}	1	0.5	2
	bC_5H_{11}	1	0.5	2
2M2B	bC_5H_{11}	1	0.5	2
	cC_5H_{11}	1	0.5	2
3M1B	cC_5H_{11}	1	0.5	2
	dC_5H_{11}	1	0.5	2
